# Spray Pyrolysis Technique; High-*K* Dielectric Films and Luminescent Materials: A Review

**DOI:** 10.3390/mi9080414

**Published:** 2018-08-19

**Authors:** Ciro Falcony, Miguel Angel Aguilar-Frutis, Manuel García-Hipólito

**Affiliations:** 1 Departamento de Física, CINVESTAV, Apdo. Postal 14-470, Delegación Gustavo A. Madero, Mexico City C.P. 07000, Mexico; 2 Instituto Politécnico Nacional, Centro de Investigación en Ciencia Aplicada y Tecnología Avanzada, Legaría 694 Colonia Irrigación, Mexico City C.P. 11500, Mexico; mafrutis@yahoo.es; 3 Instituto de Investigaciones en Materiales, UNAM, Apdo. Postal 70-360, Delegación Coyoacán, Mexico City C.P. 04150, Mexico; maga@unam.mx

**Keywords:** spray pyrolysis technique, dielectric materials, luminescent materials

## Abstract

The spray pyrolysis technique has been extensively used to synthesize materials for a wide variety of applications such as micro and sub-micrometer dimension MOSFET´s for integrated circuits technology, light emitting devices for displays, and solid-state lighting, planar waveguides and other multilayer structure devices for photonics. This technique is an atmospheric pressure chemical synthesis of materials, in which a precursor solution of chemical compounds in the proper solvent is sprayed and converted into powders or films through a pyrolysis process. The most common ways to generate the aerosol for the spraying process are by pneumatic and ultrasonic systems. The synthesis parameters are usually optimized for the materials optical, structural, electric and mechanical characteristics required. There are several reviews of the research efforts in which spray pyrolysis and the processes involved have been described in detail. This review is intended to focus on research work developed with this technique in relation to high-*K* dielectric and luminescent materials in the form of coatings and powders as well as multiple layered structures.

## 1. Introduction

The spray pyrolysis technique is a low-cost, non-vacuum required, way to synthesize materials in the form of powders and films. In the case of films, they are usually deposited over a wide variety of substrates that can be easily adapted for large area deposition and industrial production processes [1,2,3,4,5,6,7]. A large amount of the work reported using this technique is concerned with semiconductors, metal and transparent conductive oxides (TCO’s) related to their electrical conductivity characteristics. In particular, in the case of TCO’s and their relevance for photovoltaic applications, a considerable amount of effort was set to optimize their optical transparency in the visible and electrical conductivity characteristics. This was the case for indium-tin oxides (ITO), indium-Zinc Oxide (IZO), fluorinated-tin oxide (FTO) and many others [1,3]. It was until a few decades ago that different metal oxide and compounds, mixed or in a multiple layer form, incorporating a large variety of dopants were synthesized by this technique for other application purposes [8,9,10]. Thus, coatings were developed to modify the optical absorption/transmittance, and emissivity of flat glass for the automotive, as well as construction industries. Furthermore, they were also developed for multiple layered structures, such as planar waveguides and resonant optical cavities for photonics [11,12,13], as well as semiconducting and metal oxide layers. These were doped with a variety of atomic and molecular centers, synthesized by this technique, for the development of light emission devices [14,15]. The dielectric characteristics of many metal oxides were also evaluated for high dielectric constant coatings for dielectric gate layers that might find applications in MOSFET technology, as well [16,17,18,19,20].

This technique is an atmospheric pressure chemical synthesis of materials, in which a precursor solution of chemical compounds in the proper solvent is sprayed through a furnace. In the case of powders, or on a hot substrate in the case of films, where a pyrolysis reaction is achieved, metal oxides is the preferred compound to be obtained by this technique. Nonetheless, metal and semiconductor materials have also been synthesized by a proper deposition ambient and carrier gas choice [1,15]. In this paper, a revision of the work involving the spray pyrolysis technique (published in the later period of time) will be presented. This focuses on the high-*K* dielectric and luminescent properties of coatings and powders as well as multiple layered structures. This review will begin with a brief general description of the basic physical and chemical principles utilized by this technique, and the different experimental arrangements and deposition regimes that are involved in this process. The main characteristics of high-*K* dielectric materials deposited on different type of substrates will then be discussed, as well as the luminescent characteristics of both powders and coatings of materials obtained by the incorporation of dopants in a suitable matrix.

## 2. Spray Pyrolysis as Materials Synthesis Technique

The spray pyrolysis technique involves three major process stages: Precursor solution composition, aerosol generation and transport, and synthesis process. Every one of these stages is tuned according to of the final chemical and physical characteristics of the material targeted; these adjustments and the choice of materials/processes at each stage will affect the rest of the stages, to some extent. Thus, at the first stage, the chemical composition of the precursor solution will have to involve a compound(s) that will render after the pyrolysis stage the chemical composition required. The selection of the solvent will limit the maximum concentration of the precursor compound in the solution and will determine the best choice for the aerosol generation/transport process and the temperature and rate of synthesis. At the second stage, the aerosol droplet size distribution, determined by the aerosol generation mechanism, will set the morphological characteristics of the final material produced, as well as the proper range of synthesis temperatures. The carrier gas nature and flux rate will propitiate or reduce the probability of a reactive interaction with the precursor compound. At the last stage, the decision whether the final chemical reaction takes place on a gas phase or on a hot substrate will determine if the material synthesized is a powder or a film coating. In general, given an experimental setup, the synthesis parameters that are more relevant are the concentration molarity of the precursor solution, the carrier gas flux rate, and the synthesis temperature.

The solvent in the precursor solution is chosen attending to the solubility of the precursor compound and on its physical properties such as density and viscosity as well as on the final byproducts that will generate and how neutral for their disposal they will be. The preferred choice is water or a mixture of water and an alcohol, which will dissolve many inorganic salts (such as chlorides, some nitrites and fluorides). Organic salts will require organic solvents that, when properly selected, could render excellent precursor solutions, especially for thin films deposition processes [4].

**Figure 1:**
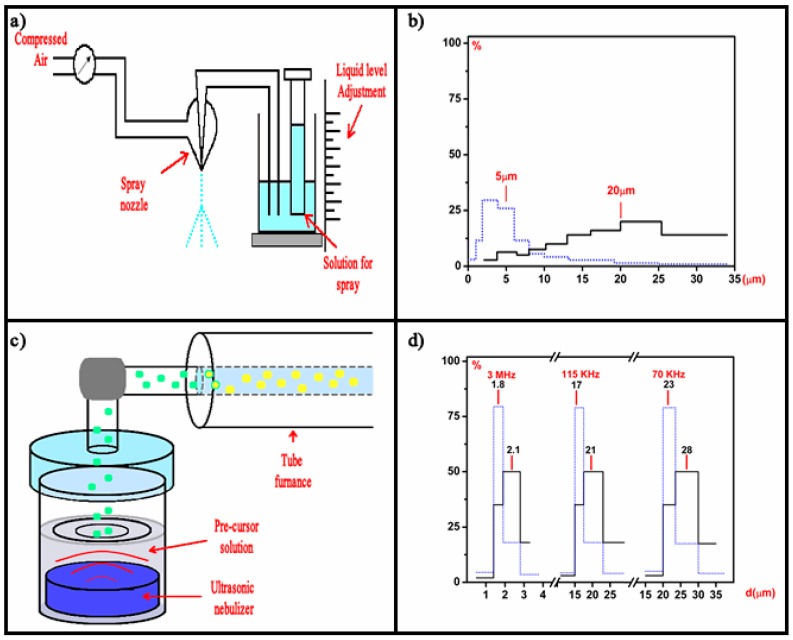
The most common aerosol generation systems, pneumatic and ultrasonic, and the droplet distribution by diameter size or by the amount of solution delivered: (**a**) Shows the pneumatic setup, and (**b**) the corresponding droplet distribution. (**c**) Shows the ultrasonic system, and (**d**) the droplet distribution for this system.

The aerosol generation mechanism could be as simple as a pneumatic system or a more complex but more tunable ultrasonic system. Figure 1 illustrates both systems. In the most common setup for a pneumatic system (Figure 1a), a Venturi nozzle is used in which the precursor solution is fed through a fine (capillary like) inlet into a pressurized carrier gas jet flow. An equation to estimate the average drop diameter has been developed for this type of nozzle [21]:

(1)  d=0.64D[1+0.011(GlGg)2][2γρυ2D]0.45 

where; *G_l_* and *G_g_* represent the mass flow rate of liquid and gas, respectively, *γ* the liquid surface tension, *ρ* the density of the gas, *D* the diameter of the spraying solution inlet orifice, and *ν* the velocity of gas. The actual experimental distribution of the droplet diameter size distribution generated by pneumatic means [3] is shown in Figure 1b, for the number of drops and for the total mass carried by the drops, in both cases a broad distribution is observed. Figure 1c shows a diagram of an ultrasonic system [5], in which the aerosol is generated by the ultrasonic waves produced with a piezoelectric disc in contact with the precursor solution. The standing waves generated at/near the surface of the liquid solution result in a generation of droplets in a combination of surface waves (capillary waves) and cavitation phenomena. The first mechanism dominates at low frequencies (20–100 kHz) and the later at frequencies above 100 kHz (0.1–5 MHz). An expression that estimates well the diameter of the droplets was derived [22] as follows:

(2)  D=(πσρf2)13+0.0013(We)0.008(Oh)−0.14n(IN)−0.28 

where

(3) $$\;We=\frac{fQ\rho }{\sigma }\;$$

(4) $$\;Oh=\frac{\mu }{f\cdot A_{m}^{2}\rho }\;$$

And

(5) $$\;I_{N}=\frac{f^{2}A_{m}^{4}}{\nu_{s}Q}\;$$

In these expressions; *f* is the ultrasonic wave frequency, *A_m_* is the amplitude, *σ* is the surface tension, *ρ* is the liquid density, μ is the viscosity and *Q* is the volumetric flow rate of the liquid and *ν_s_* is the speed of sound. Figure 1d shows the experimental distribution for the number of drops and for the total mass carried by the drops generated by ultrasound waves at three different frequencies: 0.7, 0.115, and 3 MHz. The narrow distribution of drops, as well as the control on the average size, is considered the main advantages of the ultrasonic aerosol generation systems. Once the aerosol mist is generated, it has to be transported to the material synthesis area, since in most cases the droplets in the aerosol are below 20 µm in diameter, they can be carried with a gas flow set to minimize coalescence of the drops throughout the transport process, and also to render a desired synthesis rate. Since the carrier gas will be closely present during the synthesis process, whether it is chosen to be an inert or a reactive gas becomes relevant. Thus, if the carrier gas is air, the synthesis is limited to compounds that are as stable like or even better than oxides. In the case of metals, the carrier gas has to be an inert gas which in some cases is combined with a reduction gas (N_2_ and H_2,_ as in the case of forming gas) [1]. 

**Figure 2:**
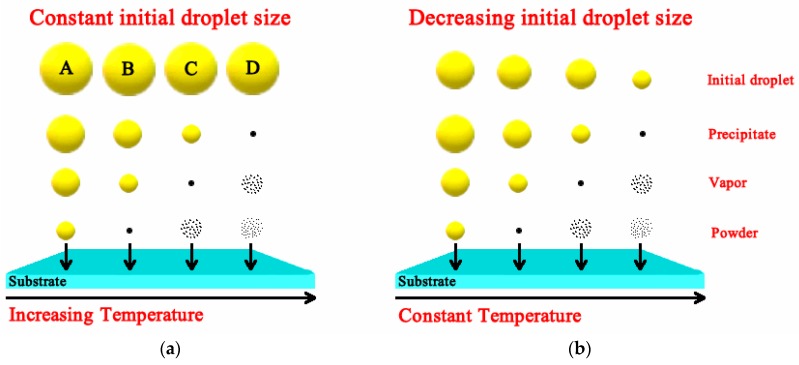
Diagram of the different process stages for the aerosol droplet evolution as it approaches the hot substrate for two cases: (**a**) Constant initial droplet size and increasing substrate temperature, and (**b**) constant substrate temperature and decreasing initial droplet size.

At the reactive zone, several parameters are determinant as to what type of material synthesis process occurs, such as temperature, droplet size and their speed. The reactive zone is, in the case of film deposition, the space near the surface of the hot substrate (a few millimeters above the surface of the substrate), or the furnace heated chamber, in the case of powder synthesis. Figure 2 shows a diagram of the different stages at which the droplet is subjected as it approaches the hot substrate for two cases a fixed droplet size and speed, different (increasing from A to D, Figure 2a) temperature of the substrate and fixed substrate temperature and speed of different droplet sizes (decreasing droplet size from A to D, Figure 2b) [23,24]. At low temperature (large initial droplet size), the solvent within the droplet is not completely vaporized and the liquid droplet hits the substrate and upon contact with it vaporizes leaving a ring-shaped dry precipitate on the substrate (process A). At low or intermediate temperature values (large or medium droplet size) the solvent is vaporized, and a dry precipitate (an amorphous precursor salt) hits the substrate surface where a pyrolysis reaction takes place (process B). At intermediate or high temperatures (medium or small droplet size) the droplet goes through all previously described stages. Near the substrate surface the dry precipitates are vaporized, propitiating a chemical vapor reaction (CVD) on the surface of the substrate (process C). Finally, for high temperature (small droplet sizes) the vaporized precipitates undergo a chemical reaction in the vapor phase before they reach the substrate surface (process D). In the case powder synthesis, similar processes occur—but for this case, the parameter that controls the occurrence of the different synthesis stages is the time of flight (time of residence) of the droplet inside the hot zone of the furnace [5]. 

## 3. High-*K* Dielectric Films

Results associated to the fabrication and characterization of high-*K* dielectrics obtained by ultrasonic spray pyrolysis (USP) is shown in this section. The synthesis of high-*K* dielectric thin films by USP is considered of great importance because, as can be inferred from the last section, the technique is neither expensive nor difficult to be developed in any fair laboratory [2]. Several high-*K* dielectrics have been attempted, including aluminum oxide thin films, zirconium oxide, and yttrium oxide [16,17,18,19,20]. The main goal of researching high-*K* dielectrics is the preparation of metal oxides that might be of interest for the scaling and gate capacitance of some devices in the future. Literature has shown clearly the need to develop high-*K* dielectric materials [25,26] for electronic microdevices in the silicon based Complementary Metal Oxide Semiconductor (CMOS) technology. This is the case of Field Effect Transistors (FETs), one of the most important devices, because of its low power consumption and performance. However, the need of down scaling has been a very dramatic issue. Furthermore, the materials involved for these applications show a dramatic constraint in the dielectric layers that play an important role in the FETs. In them, the thickness of the SiO_2_ layer needed for the gate dielectric is under 1.4 nm; so thin that the gate leakage current by direct tunneling of electrons through the SiO_2_ film becomes too high. This, and other drawbacks, have resulted in a search for better suited dielectric materials than that of SiO_2_. Since the tunneling current across a dielectric film decreases exponentially with increasing thickness ($ℑ\propto \exp\left(-2\sqrt{\frac{2m\phi }{\hbar^{2}}}z\right)$, where ϕ is the barrier height for tunneling), a thicker layer of a higher dielectric constant material than SiO_2_ is a possible solution. In a FET, the source-drain current depends on the gate capacitance: $C=\frac{k\epsilon_{0\;}A}{t}$, where $\epsilon_{0}$ is the permittivity of free space, k is the relative permittivity, A is the area and t is the oxide thickness. So, to solve the problem of leakage current due to tunneling, it is required to replace SiO_2_ with a physically thicker layer of a higher dielectric constant material. This would preserve the capacitance value with a reduced tunneling current. With this purpose in mind, the “equivalent oxide thickness”, (EOT), defined as $t_{ox}=\left(\frac{3.9}{K}\right)t_{hiK}$, where the 3.9 value is the dielectric constant of SiO_2_, has been used as a figure of merit for high-*K* dielectrics to be used instead SiO_2_. The requirements for choosing a new high K dielectric are the following: (i) Its K value must be high enough. (ii) The oxide should be thermodynamically stable when in contact with the Si channel. (iii) It must act as an insulator (large barrier with Si for both holes and electrons), and (iv) It should have a good electrical interface with Si. The static dielectric constant of high-*K* oxides is already known. Some oxides with its dielectric constant are listed in Table 1 [25,26].

### 3.1. High-K Dielectrics Materials

Some common high-*K* metal oxides that might provide thicker dielectric layers with reduced leakage (preserving the SiO_2_ equivalent capacitance values) are Ta_2_O_5_, SrTiO_3_, Al_2_O_3_ (among others). These metal oxides’ dielectric constants range from ~10 to 80, and have been employed mainly in memory capacitor applications. Among the few high-*K* materials above mentioned, Al_2_O_3_ is thermodynamically more stable when in contact with Si [26]. Before listing the synthesis and properties of aluminum oxide and other dielectrics prepared by the USP technique, it is important to realize the role of reagents and solvents involved in their synthesis.

### 3.2. The Role of the Reagents and Solvents in the USP Synthesis of High-K Dielectric Layers

The role of the reagents and solvents play a dramatic factor for achieving specific properties of films and coatings deposited by the USP technique [2]. Thus, it is important to revise the deposition process pathways, in particular, at the reactive stage (Figure 2). In the aerosol processing of materials, reactions are initiated by thermal energy. A wide number of metal-organic compounds have been used as precursors to a number of materials, via thermally induced aerosol processing. Among others, β-diketonates, carboxylates, alkoxides, and amides are frequently used. Metal β-diketonates and amides are often used as sources of metal-containing materials and frequently require reaction with an added reagent. Some metal alkoxides, β-diketonates, and amides sublimate, thus, these species are ideal and have been used for CVD like, or aerosol assisted CVD (process C in Figure 2) deposition process, that renders excellent quality layers. Metal β-diketonates have been utilized for the deposition of a large variety of materials, such as metals, metal oxides, and metal sulfides. The suggested reaction in the presence of water that produces metal oxides (MO) is as follows:

(6) $$\;M{\left(\beta -\mathrm{diketonate}\right)}_{2}+H_{2}O\to \mathrm{MO}+2H\left(\beta -\mathrm{diketonate}\right)\;$$

Organometallic compounds have been used extensively in the gas-phase synthesis of materials, particularly CVD and gas-to-particle conversion, because these compounds are often sufficiently volatile. In addition to the appropriate choice of an appropriate source of the metal, the selection of an appropriate solvent is also important. Three basic requirements should be accomplished by a solvent for its suitability in the case of ultrasonic generation of an aerosol. Firstly, the solubility of the acetylacetonates or the organometallic compounds used. The second requirement is, because of the requirement that the aerosol droplets should arrive near the substrate surface preferably in liquid state, that the solvent used should have a relatively high boiling point. The third requirement is that the solvent should also possess a low grade of viscosity to enable proper ultrasonic excitation and aerosol generation [11]. Atomization of acetylacetonates (dissolved in organic solvents) by ultrasonic excitation has been used by G. Blandenet et al. to deposit films of Al_2_O_3_, Y_2_O_3_, ZrO_2_ and other coatings on glass and stainless steel [3].

The physical properties of a few solvents used during the deposition of some high-*K* dielectric coatings in this work and in for other authors are listed in Table 2. In this review, it is highlighted the use of Dimethylformamide and a few alcohols [11].

### 3.3. Synthesis of Al_2_O_3_ Thin Films by USP Technique

Aluminum oxide thin films (Al_2_O_3_) have good thermal conductivity, low permeability to alkali ions, excellent hardness, high radiation resistance, high refractive index, high transparency, resistant against hostile environments and high dielectric constant [9,20,27,28,29]. This latter property is highly important to possibly replacement of SiO_2_ as a H-K dielectric for the microelectronic devices applications. The fabrication of aluminum oxide thin films using the USP technique has been reported, at least, since the 1980s. G. Blandenet et al. deposited Al_2_O_3_ coatings on glass by ultrasonic spraying using aluminum isopropoxide, as source of aluminum, and butanol as solvent [3]. A lot of work has been carried out since then to get this type of films with improved characteristics. Actually, the research in these films continues up to date, using the same technique and/or a related spray pyrolysis technique. It is worth to mention the recent work of B.P. Dhonge et al. [30]; or the work of A.B. Khatibani et al., who also obtained alumina thin films by spray pyrolysis [31]. In particular, excellent quality aluminum oxide thin films have been deposited using aluminum acetylacetonate, dissolved in *N*,*N*-dimethylformamide as spraying solution [9,19,20]. These work shows the versatility of USP technique and how the experimental conditions of synthesis can be optimized to get the films with the required optical, structural and dielectric properties. A few highlights of these results are described below. 

### 3.4. Experimental Details

The most appropriate reagent and solvent were found to be: Aluminum acetylacetonate (Al(acac)_3_) as source of aluminum, and *N*,*N*-dimethylformamide (DMF) as solvent. Several solutions of Al(acac)_3_ in DMF were prepared. The solutions prepared consisted in dissolving 1, 3, 5, 7, 10 and 12 g of Al(acac)_3_ in 100 mL of DMF. The versatility of the spray pyrolysis process permits the generation of aerosol streams with different reagents and/or additives that can be supplied simultaneously during the synthesis of a thin film (for example, binary oxides of some semiconductors; such as CuCrO_2_ have been deposited within this approach [32]). In the present case, a parallel aerosol stream of water mist to the aerosol of the Al(acac)_3_ in DMF solution was supplied during the synthesis of the Al_2_O_3_ thin films. The motivation to use a water aerosol was realized by the report of J.S. Kim et al. [33], who used the addition of a water mist for fabricating thin films by the CVD method. The Al_2_O_3_ films were deposited on n-type silicon wafers of low and high resistivity (0.1 and 200 Ω·cm, respectively), and on quartz slides for the optical absorption measurements. The deposition of the films was achieved at different substrate temperatures: 450, 500, 550, 600 and 650 °C. The high deposition rate of the films led to get the film thicknesses, within a few seconds or minutes, in the range of 90–130 nm. MOS (Metal-Oxide-Semiconductor) structures were fabricated with these films by thermally evaporating aluminum contacts (1.1 × 10^−2^ cm^2^) on top of the aluminum oxide thin film deposited on the silicon substrates [9,19,20]. The films resulted transparent in the whole visible range of the electromagnetic spectrum. The optical band gap of these films (about 5.63 eV) compared favorably to the best quality films obtained by other techniques. The films were found to be mainly amorphous in all cases. The films deposited with water mist showed a higher index of refraction, in contrast to the films deposited without water mist. These results might indicate that the films deposited by water mist show a higher specific density and were confirmed by the electrical response of the films, since the MOS structures fabricated with this type of films showed the best dielectric characteristics. The role of water during deposition process was perhaps to collect and remove the residual carbon from the acetylacetonate decomposition, reducing in this way the total amount of carbon and impurities that might remain in the oxide film [9,19,20]. 1 MHz and quasi-static capacitance versus voltage characteristic of the MOS structures were used to determine the density of interface states that was found in the range of 10^11^ eV^−1^·cm^−2^. This density of interface states compared favorably to other dielectric layer used in many microelectronic applications. The current density measured by the ramp I-V characteristic curves in these MOS structures, at electric fields below 2 MV/cm, was in the range of the displacement current generated by the voltage ramp applied to the MOS structure 10^−9^ Amp/cm^2^. At electric fields higher than 2 MV/cm a real current injection across the aluminum oxide (produced by Fowler-Nordheim tunneling) increases up to 10^−6^ Amp/cm^2^ at approximately 5 MV/cm without any destructive breakdown of the films [9,20]. In addition, aluminum oxide thin films as thin as 30 nm were deposited by means of a pulsed spraying setup with excellent properties [19]. This last feature showed that the ultrasonic spraying is also capable of depositing extremely thin films of aluminum oxide preserving its excellent dielectric properties.

### 3.5. Y_2_O_3_ and ZrO_2_ Films

Other dielectrics have also been considered. In particular, yttrium, zirconium and silicon oxides (Y_2_O_3_, ZrO_2_ and SiO_2_) deposited by spray pyrolysis. Even though SiO_2_ is not a high-*K* dielectric, this type of film has been obtained successfully using the spray pyrolysis technique [18]. Other high-*K* dielectrics, such as yttrium oxide thin films, have been deposited on silicon substrates using yttrium acetylacetonate as source of yttrium, and *N*,*N*-dimethylformamide as solvent. For this system, a solution of H_2_O-NH_4_OH was sprayed in parallel during the deposition process to improve the optical, structural and electrical properties of the deposited films. In this case, the films were deposited at temperatures in the range from 400 to 550 °C. The effective index of refraction measured in the films was about 1.86, and an average deposition rate ~0.1 nm/s. A highly textured surface of the films was obtained to (400) orientation. The growth of a SiO_2_ layer sandwiched between the yttrium oxide and the Si substrate was also noticed and it seemed to improve a lower interface state density, in the range of 10^10^ eV^−1^·cm^−2^. An effective dielectric constant up to 13, as well as a dielectric strength in the range of 0.2 MV/cm was obtained in a 100 nm thick film incorporated in a MOS structure. For this system, it seemed that the polycrystalline nature of these films results in a deterioration of the dielectric properties by reducing the threshold voltage needed for conduction current across the films [16,34]. Another high-*K* dielectric that has been studied is zirconium oxide (ZrO_2_). ZrO_2_ thin films were also deposited on silicon substrates by spray pyrolysis, in the temperature range from 400 to 600 °C. The use of zirconium acetylacetonate as source of zirconium and *N*,*N*-dimethylformamide was also used in this case. The films resulted with an index of refraction in the range of 2.12. The dielectric constant was about 12.5–17.5. In the best case, the films could stand an electric field up to 3 MV/cm, without presenting evidences a dielectric breakdown. Transmission electron microscopy measurements indicated that the films of ZrO_2_ were constituted by nano-crystals embedded in an amorphous matrix [16].

In summary, high quality aluminum, yttrium and zirconium oxide thin films have been deposited by spray pyrolysis using acetylacetonates dissolved in *N*,*N*-dimethylformamide. In the case of aluminum oxide, they were obtained with excellent homogeneity to thicknesses down to 30 nm. The addition of a parallel stream of water mist into the spraying solution aerosol during the deposition process resulted in a dramatic effect over the refractive index and on the dielectric characteristics of the deposited aluminum oxide films. The density of states in the range of 10^11^ eV^−1^·cm^−2^ and a destructive electric breakdown field larger than 5 MV/cm, were obtained on MOS structures fabricated with these films. Yttrium and Zirconium oxide thin films showed a higher dielectric constant than those of aluminum oxide, but lower dielectric strength, likely due to the polycrystalline nature of the films.

## 4. Luminescent Materials

Luminescent materials, in the form of powders (phosphors) and films, have been extensively studied in recent decades [35,36] because their great importance for a wide variety of applications such as: Lighting, image displays, signaling, lasers, medical applications, etc. [37,38]. They have been synthesized through a variety of physical and chemical techniques, including: Hidrothermal/Solvothermal [39,40,41], solid-state reaction [42], sol-gel [43,44], laser ablation [45,46], sputtering [47], Pechini Method [48], plasma electrolitic oxidation [49], conventional melt-quenching method [50,51], combustion synthesis [52], solvent evaporation method [53,54,55], and co-precipitation process [56]. Among these techniques, spray pyrolysis began to be used for this purpose in the mid-1980s, and it is still used today [57,58]—proving to be a practical, low cost, easy to extrapolate for large area deposition technique. In this review, an account is made on diverse luminescent materials synthesized by this technique. These materials in general involve one or more luminescent centers incorporated as dopants in a host lattice. A great variety of host lattices have been used for the synthesis (by means of spray pyrolysis) of phosphors and luminescent films, among them stand out metal oxides such as: ZrO_2_, Al_2_O_3_, HfO_2_, Y_2_O_3_, ZnO, In_2_O_3_, ZnSiO_3_, CdO, (Y, Gd)BO_3_, Gd_2_O_3_, LaPO_4_, BaMgAl_10_O_17_, and some sulfur based compounds such as: ZnS, CaSO_4_, CdS, and others. The luminescent active centers have been mainly RE (Rare Earth) and some transition metal ions. In some cases, luminescence emission has been observed to be generated by mechanisms that involve structural defects and intrinsic states in the host lattices as well. This review focuses mainly on the work done on host lattices such as: ZrO_2_, Al_2_O_3_, HfO_2_, Y_2_O_3_, ZnO, and ZnS with different dopants.

### 4.1. ZrO_2_

Virtually before 1999, ZrO_2_ had not been used as a host lattice to produce phosphor materials synthesized by spray pyrolysis technique. In that year, some results were reported about photoluminescence (PL) and thermoluminescence (TL) properties of ZrO_2_:Tb^3+^ films deposited by a pneumatic spray pyrolysis (PSP) system [59]. These films excited by 275 nm exhibited four peaks at 487, 542, 582, and 619 nm—typical of electronic transitions in the Tb^3+^ ions. The TL glow curve displayed two peaks at 112 °C and 270 °C for the ZrO_2_:Tb^3+^ films exposed to 260 nm UV radiations. In addition, the TL response was linear in the range of 40 to 240 mJ·cm^−2^ spectral UV irradiance. These results exhibited that ZrO_2_:Tb^3+^ films had appropriate characteristics for their use as a UV dosimeter as well as PL phosphor. In a later investigation (2001) on this material [60], a deeper analysis was made on the thermoluminescence mechanisms. Two important parameters in TL studies such as activation energy (E) and the frequency factor (S) were investigated. In this contribution, the Lushchik and Chen methods were used to determine the kinetic parameters which showed second order kinetics for both the first and second glow TL peaks. 

Furthermore, in 2001, PL and cathodoluminescence (CL) feature of ZrO_2_:Tb^3+^ films, deposited by the PSP, technique was reported [61]. In this case different deposition parameters, such as substrate temperatures, doping concentrations, and the flow of the precursor solution, were studied. Substrate temperatures higher than 400 °C rendered a polycrystalline material with metastable tetragonal or cubic phases. With increasing deposition temperatures, the PL and CL emission intensities (excited with 250 nm light) also increased. The PL and CL emission spectra showed the characteristic peaks associated with the electronic transitions of Tb^3+^ ions. Concentration quenching for the PL and CL emissions occurred at doping concentration greater than 1.96 and 1.17 at.%, respectively. Similar studies were conducted on ZrO_2_:Eu^3+^ films [62]. Depending on the substrate temperature, these films were amorphous or polycrystalline (tetragonal-cubic phase). A strong red emission was observed which was generated by the ^5^D_0_ → ^7^F_2_ transition typical of the Eu^3+^ ions. From those studies, it became clear that zirconia was a suitable host lattice for RE ions.

For the first time, a study on luminescent emissions from ZrO_2_: Mn^2+^ films deposited by the USP technique was reported in 2002 [63]. These films were deposited at substrate temperatures ranging from 250 to 500 °C. The PL and CL (7 KeV) emission spectra showed a broad band (450–750 nm) centered at 650 nm (red), which is associated with the electronic transitions ^4^T_1_(^4^G) → ^6^A_1_(^6^S) of the Mn^2+^ ions. A decrease of the luminescence, as a function of the doping concentration, substrate temperature and electron accelerating voltage was observed. The maximum emission intensity was observed for films deposited at 250 °C, EDS measurements showed that these films had a high amount of incorporated chlorine (from the precursors in the spraying solution), which acts as a co-activator for the red emission. As the deposition temperature increased, the amount of chlorine in the film (as well as the red luminescence emission intensity) decreased. The presence of chlorine was necessary for the red luminescence emission to occur. CL spectra obtained at higher electron accelerating voltages (10 KeV) from samples deposited at 500 °C showed, instead of the red emission, a wide band centered at 590 nm (yellow)—which is also characteristic of Mn^2+^ ions.

ZrO_2_:Eu^3+^ phosphors consisting of spherical, dense and sub-micrometer size particles were successfully synthesized by the USP technique in 2005 [64]. The X-ray diffraction (XRD) measurements indicated that the crystallinity of these powders increased with increasing postdeposition annealing temperature. Several characterization techniques were used to study this material: Including PL emission spectra, and decay time measurements. The excitation spectrum showed a band centered at 248 nm corresponding to a charge transfer transition from Eu-O generated electronic states in the ZrO_2_ host matrix. The emission spectra exhibit the typical (red) bands of Eu^3+^ ions. The optimal concentration of Eu^3+^ ions was 10 at.% and it was observed that the spherical morphology of the particles improves the intensity of the PL emission.

A research work on ZrO_2_:Pr^3+^ films was published in 2007 [65]. In this case, PL and CL properties were studied as a function of growth parameters such as the substrate temperature and the Pr^3+^ ions concentration. XRD studies indicated a tetragonal crystalline structure for zirconia as the substrate temperature was increased. The PL spectra exhibited bands centered at 490, 510, 566, 615, 642, 695, 718, 740 and 833 nm; associated with the electronic transitions ^3^P_0_ → ^3^H_4_, ^3^P_0_ → ^3^H_4_, ^3^P_1_ + ^1^I_6_ → ^3^H_5_, ^1^D_2_ → ^3^H_4_, ^3^P_0_ → ^3^H_6_, ^1^D_2_ → ^3^H_5_, ^1^D_2_ → ^3^H_5_, ^3^P_0_ → ^3^F_3*,*4_, and ^1^D_2_ → ^3^F_2_ of the Pr^3+^ ions. As the substrate temperature was increased, an increasing intensity of the PL emission was observed. Also, a quenching of the PL and CL emissions, with increasing doping concentration, was detected. Interestingly the CL spectra, as a function of the electron accelerating voltage, showed an evolution of the highest peak: For low electron accelerating voltages (4 kV) the red emission (615 nm) is the maximum, and for high voltages (15 kV) the most intense band is the blue (around 490 nm). 

The cathodoluminescence properties of ZrO_2_:Er^3+^ films were reported in 2014 [66]. These films were deposited at different temperatures from 400 °C up to 550 °C. As substrate temperatures are increased, the films showed a tetragonal phase. CL emission spectra showed bands centered at 524 (green), 544 (green) and 655 (red) nm associated with the electronic transition ^2^H_11/2_ → ^4^I_11/2_, ^4^S_3/2_ → ^4^I_15/2_, and ^4^F_9/2_ → ^4^I_15/2_ of Er^3+^ ions. The highest emission intensity is achieved in samples deposited at 500 °C doped with 5 at.% of Er^3+^ ions. Also, the CL emission intensity increases as the substrate temperature and electron accelerating voltage values increase.

Investigations on ZrO_2_:Dy^3+^ and ZrO_2_:Dy^3+^+*x*Li^+^ films were published in 2015 [67]. XRD measurements, as a function of the deposition temperature, indicated a meta-stable tetragonal crystalline structure of the zirconia. PL and CL features of the films were studied as a function of synthesis parameters such as the substrate temperature and the Dy^3+^ and Li^+^ concentrations. All luminescent emission spectra showed peaks located at 485 (blue), 584 (yellow), 670 (red) and 760 nm; which correspond to electronic transitions ^4^F_9/2_ → ^6^H_15/2_*,* ^4^F_9/2_ → ^6^H_13/2_, ^4^F_9/2_ → ^6^H_11/2_, and ^4^F_9/2_ → ^6^H_9/2_, of Dy^3+^, respectively. The Li^+^ incorporation in the ZrO_2_:Dy^3+^ films produced an improvement in the intensity of the luminescent emission, presumably because it acts as a charge compensator and because it contributes to improving the crystalline structure of the host lattice. The CIE color coordinates (0.3475, 0.3609) of these films were found within the warm white light emission region. These spectroscopic characteristics allowed to propose this material for application in solid-state lighting (SSL), especially for white lighting emission applications. It is observed that, as the concentration of Li^+^ ions increases, they come closer to the perfect white area of the CIE color coordinates (0.3333, 0.3333). 

Moreover, in 2015 a work on ZrO_2_, ZrO_2_:Dy^3+^ and ZrO_2_:Dy^3+^ + Gd^3+^ films was published [68]. The synthesis and the characterization conditions were carried out as described in Reference [67]. The relative concentrations of Dy^3+^ and Gd^3+^ ions were varied; the emission spectra of these films exhibited bands in the blue and yellow regions. The incorporation of Gd^3+^ ions in ZrO_2_:Dy^3+^ films generated a remarkable increase in the intensity of the luminescent emission (approximately 15 times). In principle, the host lattice absorbs the excitation energy which is transferred to the Gd^3+^ ions which in turn transfers it to the Dy^3+^ ions. The CIE chromaticity diagram exhibited a cold-white emission (Dy^3+^-Gd^3+^ doped samples) and a warm-white emission (Dy^3+^ doped samples), which shows the potential of these films for generating white light coatings for solid state lighting (SSL) applications. 

The PL and structural properties of co-doped ZrO_2_: Eu^3+^ + Tb^3+^ films, were also reported in 2015 [69]. The PL spectra showed the typical emission bands associated with the Tb^3+^ and Eu^3+^ ions, as well as a broad emission, peaked at 440 nm associated to radiative transitions within the ZrO_2_ host lattice. These films displayed multicolored emissions depending of the ratio Eu^3+^/Tb^3+^ and the excitation wavelength. The observed colors were: Blue (from the host lattice), green (from the ZrO_2_:Tb^3+^ films), red-orange (from the ZrO_2_:Eu^3+^ films), yellow (from the ZrO_2_:Eu^3+^ + Tb^3+^ films, excited with 288 nm) and bluish-white and yellowish white (from the ZrO_2_: Eu^3+^ + Tb^3+^ films, excited with 368 or 380 nm). The CIE coordinates of the double-doped ZrO_2_:Tb^3+^ (10 at.%) + Eu^3+^ (5 at.%) films lie in the white light region of the chromaticity diagram and show good potential for lighting devices and photonic applications.

### 4.2. Al_2_O_3_

A pioneering work on luminescent Al_2_O_3_:Tb^3+^ films appeared in 1992 [70]. The films were deposited by the PSP technique on either plain or conductive oxide coated glass substrates at deposition temperatures in the range of 270–450 °C. PL emission from these films showed well-defined peaks at 490 and 550 nm, which were associated to the electronic transitions corresponding to Tb^3+^ ions. The relative emission intensity was strongly dependent on the type of substrate, the deposition temperature and the amount of Tb^3+^ ions incorporated in the films. Two years later, an investigation on Al_2_O_3_:CeCl_3_ films was published in 1994 [71]. PL spectra (excited with 300 nm light) showed a broad emission formed by two overlapping peaks at 365 and 395 nm. It was suggested that these bands originate from the 5d to 4f electronic energy levels of Ce in the CeC1_3_ molecule. The PL emission intensity of these peaks was strongly dependent on the doping concentration and the substrate temperature. The films with greater intensity were those deposited at the lowest temperature, where there is a greater amount of CeCl_3_ incorporated in the films. As the temperature increases, the concentration of CeCl_3_ molecules decreases and so does the PL emission intensity—therefore, the presence of this molecule is essential for an optimal emission of blue light. Also, a quenching of the PL is observed for CeCl_3_ concentrations higher than 1 at.%. Another research on Al_2_O_3_:Eu^3+^ films was published in 2000 [72]. These films were deposited by the USP technique at substrate temperatures from 300 to 540 °C and the Eu^3+^ doping concentration was varied. All films were amorphous in structure and the PL spectra were measured as a function of substrate temperature and doping concentration. The excitation spectrum showed an intense peak centered at 395 nm. All the PL emission spectra (excited by 395 nm) showed bands located at 587, 600, 612, and 648 nm—typical of the electronic transitions in Eu^3+^ ions. It was observed a concentration quenching of the PL emission intensity at values of above 1.5 at.% in the films. Thus, it was shown that Al_2_O_3_ is a suitable host lattice to support RE ions (such as Eu^3+^) to generate strong PL emissions.

In 2003, a new research in Al_2_O_3_:Tb^3+^ films was published [73]. In this case, the transparency of the films was up to 88% on the 400 to 700 nm range. These was possible because the use of organic source reactive for both aluminum and terbium (acetylacetonates) that were dissolved in dimethylformamide and sprayed, deposited at temperatures up to 600 °C. These films were mostly amorphous in the range of deposition temperatures studied with an average roughness of 14 Å or less; which was perfect for the design and development of microdevices integrating this type of films. PL and CL spectra, studied as a function of the deposition parameters such as doping concentrations and substrate temperatures, were typical of the transitions among the electronic energy levels of the Tb^3+^ ions. Thus, from this work, it is clear that the use of acetylacetonates as precursors, generates the formation of high transmittance films with low roughness, as described in the dielectric section thin films, in contrast to those films synthesized from chlorides, nitrates or acetates (dissolved in water) which are, in general, very rough and opaque. 

An energy transfer mechanism between Ce^3+^ and Mn^2+^ ions in alumina films was reported in 2005 [74]. Blue and red light emitting Al_2_O_3_:Ce^3+^:Mn^2+^ films, under ultraviolet light excitation, were investigated in this case. The blue emission is due to transitions from the excited state 5d to the split ground state ^2^F of the Ce^3+^ ions. The usually weak Mn^2+^ ions red emission, attributed to intra 3d transitions, was enhanced by an efficient energy transfer from the Ce^3+^ ions. The energy transfer mechanism was an electric dipole–quadrupole interaction with a quantum efficiency estimated to be near to 100%, which makes these films interesting phosphors for the design of microdevices based on luminescent layers in flat-panel displays. Other studies on this type of amorphous Al_2_O_3_:Ce^3+^:Mn^2+^ films were also published [75,76]. However, in this case, the precursors were AlCl_3_, CeCl_3_ and MnCl_2_ dissolved in deionized water (Ce: 10 at.%; Mn: 1, 3, 5, 7 and 10 at.%), deposited at a substrate temperature of 300 °C. The chemical composition and the profile distribution of the dopant ions across the films were determined by Rutherford backscattering (RBS). A homogeneous depth profile of both Ce^3+^ and Mn^2+^ ions was found within the films, and the overall measured quantities were as expected from the solution concentrations. Chlorine, which plays a significant role in luminescent properties, was detected in important quantities, something that was expected due to the low deposition temperatures used in this case. The red emission from manganese-doped samples was strongly enhanced with the co-doping with Ce due to the efficient energy transfer mechanism from Ce^3+^ to Mn^2+^ ions. From XPS analysis, it was determined that a considerable amount of Mn ions remains linked to chlorine, while Ce is mostly in an oxidized state.

In 2010, alumina was used to host three ions (Tb^3+^, Ce^3+^_,_ and Mn^2+^) to generate white light when excited by ultraviolet light [77]. These amorphous films were also deposited at 300 °C. Sensitization of Tb^3+^ and Mn^2+^ ions by Ce^3+^ ions gave rise to blue, green and red luminescent emission when the film was excited with UV radiation. The overall efficiency of such energy transfer was about 85% upon excitation with 312 nm light. Energy transfer from Ce^3+^ to Tb^3+^ ions through an electric dipole–quadrupole interaction mechanism appeared to be more probable than the electric dipole–dipole one. A strong white light emission from the Al_2_O_3_:Ce^3+^ (1.3 at.%):Tb^3+^ (0.2 at.%):Mn^2+^ (0.3 at.%) films under UV excitation was obtained. The high efficiency of energy transfer from Ce^3+^ to Tb^3+^ and Mn^2+^ ions, resulted in a cold white light emission (*x* = 0.30 and *y* = 0.32). Thus, these films resulted interesting material for the design of efficient UV pumped phosphors for white light generation which could be integrated in light emitting microdevices. 

Similarly, alumina co-doped with Dy^3+^ and Ce^3+^ ions was reported in 2011 [78]. The PL properties of these films were studied through excitation, emission spectra measurements and decay time spectroscopy. These films emitted a combination of blue and yellow colors through an efficient energy transfer (77%) from Ce^3+^ to Dy^3+^ ions. It was inferred that such energy transfer was non-radiative, taking place between Ce^3+^ and Dy^3+^ clusters, through a short-range interaction mechanism. Ce^3+^ doped single films emitted in the violet-purplish-blue region; whereas co-doped films the presented a cold-white light emission. The PL properties of tri-doped Al_2_O_3_:Ce^3+^:Dy^3+^:Mn^2+^ films were published in 2012 [79]. Nonradiative energy transfer from Ce^3+^ to Dy^3+^ and Mn^2+^ was reported upon UV excitation at 278 nm. From lifetime data, it was deducted that the energy transfer was nonradiative in nature. Simultaneous emission of all co-dopant ions in the blue, yellow and red regions, resulted in white light emission with CIE 1931 chromaticity coordinates, *x* = 0.34 and *y* = 0.23, with a color temperature of 4900 K. Thin films as these might contribute to the development of materials that, pumped with AlGaN-based LEDs, could generate white light emission.

Also, in 2012, a study on the PL characteristics, under continuous and pulsed excitation of Eu-doped alumina films was reported [80]. It was determined that localized states in the undoped Al_2_O_3_ host lattice, excited with 250 nm radiation, emit a violet color (broad band centered at 415 nm) associated to a radiative recombination process involving F centers. When Eu^3+^ ions were incorporated into these films, a charge transfer mechanism to these ions from the localized states seems to occur predominantly. The Eu^3+^ related emission, generated in this way, results intensified and luminescence decay time extended as compared to that obtained when the excitation is achieved through an inter-electronic energy level transition in the Eu^3+^ ion, excited by 395 nm radiation. 

Subsequently, in 2013, a contribution on the white light emission from Al_2_O_3_:Ce^3+^:Tb^3+^:Mn^2+^ and HfO_2_:Ce^3+^:Tb^3+^:Mn^2+^ films was published [81]. These oxide films doped with CeCl_3_/TbCl_3_/MnCl_2_ were deposited at 300 °C. XRD measurements exhibited a very broadband typical of non-crystalline materials. Non-radiative energy transfer from Ce^3+^ to Tb^3+^ and Mn^2+^ ions is observed upon UV excitation at 280 nm; the energy transfer could take place in Ce^3+^-Tb^3+^ and Ce^3+^-Mn^2+^ clusters through an electric dipole-quadrupole interaction mechanism. This energy transfer gives place to a simultaneous emission of the donor and acceptor ions in the blue, green, yellow and red regions, resulting white light emission. The chromaticity coordinates for Al_2_O_3_:Ce^3+^:Tb^3+^:Mn^2+^ films and color temperatures were: (0.30, 0.32) and 7300 K (cold-white color). The chromaticity coordinates for HfO_2_:Ce^3+^:Tb^3+^:Mn^2+^ films and color temperatures were (0.32, 0.37) and 6000 K (warm-white color).

Another study on PL emission (white emission) from single and double layered Al_2_O_3_:Ce^3+^:Tb^3+^:Eu^3+^ films was presented in 2013 [10]. These films were deposited using acetylacetonates (dissolved in dimethylformamide) as precursors. Eu^3+^ and Tb^3+^ doped films showed the typical emissions of these trivalent ions (red and green, respectively). Ce doped films showed two broad bands associated with the 5d to 4f transitions of the Ce^3+^ ion, centered at ~400 and 510 nm. As expected from films deposited with organic precursors, these films had low surface roughness (lower than 3 nm) and thicknesses between 50 and 260 nm. The double layer stacks involved first an Eu^3+^ doped film followed by a second Ce^3+^-Tb^3+^ co-doped layer. The films were transparent in the visible region, with an optical bandgap of approximately 5.63 eV. The PL of these stacks was an overlap of the emissions corresponding to all the dopants when excited with 300 nm light, resulting in an intense white light emission, which would be suitable for the design of electroluminescent microdevices.

The PL characteristics of Eu^3+^ doped alumina films co-doped with Bi^3+^ and Li^+^ were published in 2015 [82]. In this case, the incorporation of Bi^3+^ and Li^+^ ions as co-dopants in Al_2_O_3_:Eu^3+^ films and its effect on the luminescence characteristics of this material were described. Both Bi^3+^ and Li^+^ do not introduce new luminescence features but affect the luminescence intensity of the Eu^3+^ related emission spectra as well as the excitation spectra. The introduction of Bi^3+^ generates localized states in the aluminum oxide host that result in a quenching of the luminescence intensity, while Li^+^ and Bi^3+^ co-doping increases the luminescence intensity of these films. It was found that the Eu^3+^ ions emission intensity in these films, when Bi^3+^ ions were added together with Li^+^, produce an increase of 62% in the emission intensity. It was suggested that the role of Li^+^ co-doping was to redirect the energy paths back to the Eu^3+^ ions from the Bi^3+^ ions. Analysis of time decay measurements of the Eu^3+^ related emission in the amorphous alumina films indicated the presence of two type of sites in the short-range surroundings of the Eu^3+^ ions that could be correlated with those around this ion in α or γ Al_2_O_3_ crystalline phases.

### 4.3. HfO_2_

Luminescent HfO_2_:Mn^2+^ films (deposited by Ultrasonic Spray Pyrolysis technique) were reported for the first time, in 2004 [83]. The deposited films were amorphous at deposition temperatures up to 300 °C; for higher temperatures a polycrystalline material was obtained with a monoclinic HfO_2_ phase. The cathodoluminescence (CL) spectra showed blue–green and red bands associated with the electronic transitions ^4^T_1_(^4^G) → ^6^A_1_(^6^S) of the Mn^2+^ ions. A dependence of the CL emissions, as a function of the doping concentration, substrate temperature and electron accelerating voltage was reported. It was determined that both amorphous and polycrystalline hafnium oxide make efficient host for Mn^2+^ ions, and that the relative content of chlorine in the processed films have an important role on the luminescent emission intensity of the studied materials.

USP deposited HfO_2_:CeCl_3_ films luminescent properties were published in 2007 [84]. These films were deposited from hafnium dichloride oxide and CeCl_3_ dissolved in deionized water (18 MΩ/cm). The PL characteristics of the HfO_2_:CeCl_3_ films were studied as a function of doping concentrations and substrate temperature. XRD measurements showed the monoclinic phase of HfO_2_ for samples deposited at deposition temperatures higher than 400 °C. These films showed a violet–blue PL emission that could easily be seen with the naked eye in normal room light. Also, PL emission and excitation spectra evidence the presence of two different Ce^3+^ centers in HfO_2_. A complete concentration quenching of the luminescence of one of the two centers is observed at high concentration of CeCl_3_ (15 at.% in the start solution), which suggests a fast energy transfer from the high-energy to the low energy centers. Finally, it was confirmed that HfO_2_ is an adequate host matrix for rare earth ions as active centers to generate strong violet–blue PL emissions. 

Also, in 2007, a work on PL properties of HfO_2_:Tb^3+^films was published [85]. The PL properties of these films were studied as a function of deposition temperature and Tb^3+^ ions concentration. The films were deposited the USP technique from aqueous solution of Hafnium and Terbium chlorides. Results showed that crystalline structure of HfO_2_:Tb^+3^ films depends on the deposition temperature. PL excitation spectrum showed a wide band centered at 262 nm while the PL emission spectra showed bands centered at 488, 542, 584 and 621 nm, which correspond to the electronic transitions: ^5^D_4_ → ^7^Fj (j = 3, 4,5, 6) typical of trivalent terbium ions. The dominant emission intensity corresponds to the green color (542 nm), which depended on the terbium concentration incorporated in the host lattice; the optimum doping concentration was 5 at.% Tb^+3^ in the spraying solution. 

The PL and CL characteristics of HfO_2_:Sm^3+^ films were published in 2008 [86]. These films were deposited by the USP technique on Corning glass substrates at deposition temperatures ranging from 300 to 550 °C using chlorides as precursor materials. Scanning electron microcopy (SEM) micrographs revealed rough surfaces morphology with spherical particles. The PL and CL spectra exhibited four main bands centered at 570, 610, 652 and 716 nm, which are due to the well-known intra-4f transitions of the Sm^+3^ ions. It was found that the overall emission intensity rose as the deposition temperature was increased. Moreover, a concentration quenching of the emission intensity was observed for doping concentration higher than 0.7 at.% as measured by EDS. These films showed good adherence to the substrate and a high deposition rate of up to 2 µm per minute. In addition, The CL emission intensity was found to increase as the electron accelerating voltage was raised.

Also, in 2008, HfO_2_ films doped with CeCl_3_ and/or MnCl_2_ were deposited at 300 °C by the USP technique [87]. The XRD results revealed that the films were predominantly amorphous. HfO_2_: CeCl_3_ showed a violet-blue emission. The weak green–red emissions of Mn^2^^+^ ions was enhanced through an efficient energy transfer from Ce^3^^+^ to Mn^2^^+^ ions in the co-doped films. Spectroscopic data indicated that this energy transfer was nonradiative in nature and it could occur in Ce^3^^+^ and Mn^2^^+^ clusters through a short-range interaction mechanism. The efficiency of this energy transfer increases with the Mn^2^^+^ ion concentration, so that an efficiency of about 78% is achieved for a 5 at.% of MnCl_2_ concentration. The HfO_2_:CeCl_3_:MnCl_2_ films are interesting phosphors for the design of luminescent layers emitting simultaneously in the three primary colors: Violet-blue, green and red. 

The HfO_2_ host lattice was also used to house, simultaneously, ions such as Ce^3+^, Tb^3+^ and Mn^2+^ to generate cold white light [88]. These films were either doubly doped with CeCl_3_ and TbCl_3_ or tri-doped with CeCl_3_, TbCl_3_, and MnCl_2_ and deposited at 300 °C. In the doubly doped films, energy transfer from Ce^3+^ to Tb^3+^ ions could take place in Ce^3+^-Tb^3+^ clusters through an electric dipole-quadrupole interaction; the efficiency of this transfer was about 81% upon excitation with 270 nm light. In the triply doped films, both Tb^3+^ and Mn^2+^ ions, can be sensitized by Ce^3+^ ions. The efficiency of energy transfer from Ce^3+^ to Tb^3+^ and Mn^2+^ ions was enhanced by increasing the Mn^2+^ concentration, up to about 76% for the films with the highest Mn^2+^ ions content (1.6 at.%). The simultaneous emission of these ions under UV excitation resulted in white light luminescence. 

The PL and TL properties of HfO_2_ films were investigated [89], these films were synthesized from hafnium chloride as raw material in deionized water as solvent and were deposited at temperatures from 300 to 600 °C. SEM images showed that the film’s surface resulted very rough with semi-spherical promontories. UV irradiation was used in order to perform the thermo-luminescent (TL) characterization of these films; the 240 nm wavelength irradiation induced the best response. The PL spectra showed emission bands, centered at 425, 512 and 650 nm, associated to impurities such as chlorine and/or structural defects. As the substrate temperature was raised, a higher intensity of the band centered at 425 nm was observed. The TL experimental results showed that HfO_2_ films could be useful in UV radiation dosimetry applications, using the TL method mainly in the interval of 200–400 nm; indicating an advantage over other ultraviolet dosimeters currently used.

An investigation on the luminescent properties of HfO_2_ films co-doped with Ce^3+^ and several concentrations of Dy^3+^ was presented in 2011 [90]. The deposition temperature was 300 °C. PL emissions from Dy^3+^ ions centered at 480 nm (blue) and 575 nm (yellow) associated with the ^4^F_9/2_ → ^6^H_15/2_ and ^4^F_9/2_ → ^6^H_13/2_ electronic transitions, respectively, were observed upon UV (280 nm) excitation via a non-radiative energy transfer from Ce^3+^ to Dy^3+^ ions. Such energy transfer via an electric dipole–quadrupole interaction appeared to be the most probable transfer mechanism. The efficiency of this transfer increases up to 86 ± 3% for the film with the highest Dy^3+^ content (1.9 ± 0.1 at.% as measured by EDS). The possibility of achieving the coordinates of ideal white light with increasing the concentration of Dy^3+^ ions was demonstrated. 

The PL, CL, and TL characteristics of HfO_2_:Dy^3+^ films were also reported in 2014 [91]. The films were deposited at temperatures ranging from 300 to 600 °C, using chlorides as precursor reagents. XRD diffraction studies showed the presence of HfO_2_ monoclinic phase in the films deposited at substrate temperatures greater than 400 °C. The surface morphology of films showed a veins shaped microstructure at low deposition temperatures, while at higher temperatures the formation of spherical particles was observed. The PL (excitation = 248 nm) and CL spectra of the doped films showed the highest emission in the band centered at 575 nm (yellow) corresponding to the transitions ^4^F_9/2_→^6^H_13/2_, which is a typical transition of Dy^3+^ ions. Regarding the TL behavior, the glow curve of HfO_2_:Dy^+3^ films exhibited spectrum with one broad band centered at about 150 °C. The highest intensity TL response was observed on the films deposited at 500 °C. A concentration quenching was observed and the optimum DyCl_3_ concentration was 1 at.% in the initial solution. It was also determined that substrate temperature for the sample with maximum PL emission intensity was 600 °C. The PL (yellowish-white emission) is intense since it can be observed by the naked eyes with normal ambient illumination. 

HfO_2_ films co-doped with Tb^3+^ or Eu^3+^ ions using acetylacetonates as precursors, were studied [92]. The films presented transmittance values in the visible region ≅90% and surface roughness less than 3.9 nm. These films were polycrystalline with a monoclinic phase for films deposited at substrate temperatures higher than 500 °C. The luminescent emissions (PL and CL) were typical of Tb^3+^ and Eu^3+^ ions with a luminescence concentration quenching observed for both Tb^3+^ and Eu^3+^ ions at 5 and 10 at.%, respectively. The peak PL and CL emission intensities for single doped films were observed for HfO_2_:Tb^3+^ (5 at.%) and HfO_2_:Eu^3+^ (10 at.%) films deposited at 500 °C. The refractive index observed in these films was between 1.97 and 2.04 and an optical band gap of 5.4 eV. The PL decay time measurements was measured on some HfO_2_:Tb^3+^, Eu^3+^ samples. QE around 35% and 25% were obtained using excitation wavelengths of 204 nm for Tb^3+^ and 215 nm for Eu^3+^, respectively. HfO_2_ films co-doped with Tb^3+^ and Eu^3+^ ions were synthesized at substrate temperatures from 400 to 600 °C using chlorides as reactive source materials [93]. These films became polycrystalline at 600 °C exhibiting the HfO_2_ monoclinic phase. Tuning by the means of the excitation wavelength and the relative concentration of the co-dopants, PL spectra with several shades, from blue to yellow (including white light) were obtained due to the combined emissions of Tb^3+^ (green), Eu^3+^ (red) ions and the host lattice (HfO_2_) violet-blue emission. The best white light emission (*x* = 0.3343, *y* = 0.3406) was obtained with 382 nm excitation light and 1.35 and 0.88 at.% of Tb and Eu in the films, respectively. The CL emission spectra for these films also showed emissions from green to red (including yellow, orange, and other intermediate emissions depending on the relative content of Tb and Eu in the film). Quantum efficiency values between 47% and 78% were obtained for these films, depending on the excitation wavelength and co-doping concentrations.

### 4.4. Y_2_O_3_

The first publication on Y_2_O_3_:Eu^3+^particles (synthesized by the spray pyrolysis process) was registered in the year 2000 [94]. These particles were prepared from high solution concentrations which had a more hollow and porous structure than those prepared from low-concentration solutions. The PL spectra showed a prominent peak at 612 nm (pure red color). The colloidal seed-assisted spray pyrolysis introduced in this paper was found to be applicable to the control of morphology of phosphor particles when the stock solution concentration was high. For the colloidal seed-assisted spray pyrolysis, the stable colloidal solution should be used for homogeneity of phase and morphology of the phosphor particles. The colloidal solution of Y and Gd hydroxy carbonate sol obtained by the liquid phase reaction method, using urea, was appropriate for the preparation of Y_2_O_3_:Eu^3+^ particles of filled and non-porous structure at high concentration of the precursor solution. The fine particles size prepared from the colloidal solution compared to those of the aqueous solution also revealed that the particles prepared from colloidal solution are much less hollow. 

CL of USP deposited Y_2_O_3_ thin films doped separately with Eu^3+^, Tb^3+^ and Tm^3+^ were reported in 2001 [95]. CL spectra for films doped with Eu^3+^, Tb^3+^ or Tm^3+^ ions presented red, green, and blue light emissions, respectively. The blue emission of Y_2_O_3_:Tm^3+^ films had dominant peak at 476 nm. The CL intensity of these films depended strongly on annealing conditions and thulium doping concentration, presenting a maximum luminance of 30.4 cd/m^2^. For the Eu^3+^-doped films, a luminance of 255 cd/m^2^ was obtained with a dominant peak centered at 604 nm. The luminance for the Tb^3+^-doped film was 72 cd/m^2^ with a dominant peak at 547 nm.

The role of LiCl added as flux on the luminescence properties of USP synthesized Y_2_O_3_:Eu^3+^ phosphors was investigated in 2002 [96]. The maximum PL intensity was obtained for phosphors prepared at 1300 °C from solution with LiCl flux, their intensity was 50% higher than that of phosphors prepared from solution without flux. The PL intensities of phosphors prepared at 700 and 900 °C from flux solution were 200% and 134% of those phosphors processed from solutions without flux at the same synthesis temperatures. LiCl flux played the role of enhancing the luminescence of Eu^3+^ ions into Y_2_O_3_ host lattice by reducing defects in the phosphor particles.

Furthermore, in 2002, a study on spherical particles of Y_2_O_3_:Eu^3+^ was published [97]. Y_2_O_3_:Eu^3+^ luminescent particles of spherical shape, filled morphology, and high brightness were prepared by combination of colloidal seed assisted spray pyrolysis and flux-added spray pyrolysis. Y_2_O_3_:Eu^3+^ particles processed from Y colloidal solution with 5 at.% LiCl/KCl flux showed completely spherical shape, filled morphology, high crystallinity, and significantly improved PL emission intensity, which was 30% higher than that of particles prepared by general spray pyrolysis. 

Another study on Y_2_O_3_:Eu^3+^ powders was published in 2005 [98]. These powders were synthesized by spray pyrolysis process and annealed at several temperatures, in the range 900–1400 °C, to achieve crystallized luminescent materials. The microstructure and macrostructure of these powders were investigated by high resolution SEM images and XRD measurements. The luminescent properties were measured under VUV excitation (254 nm). The results of this work allowed to understand the influence of the phosphors’ microstructure on PL characteristics. The spray pyrolysis powder PL efficiencies excited at 254 nm were lower than that of the commercial phosphor but under a 600 mbar Ne–Xe plasma excitation (this measurement provides a characteristic close to the working conditions in plasma display panels); the powder the brightness was equal that of the commercial phosphor. The results allowed differentiating the microstructure and macrostructure influence on luminescence. Eventually, a suitable phosphor powder for plasma display panels less dense than the commercial one has been prepared by spray pyrolysis.

A control of the morphology of Y_2_O_3_:Eu^3+^ phosphor particles in the spray pyrolysis process was attempted by using citric acid and polyethylene glycol (PEG) as additives in the spray precursors [99]. Three different morphologies of phosphor particles were obtained: Smooth spheres, rods, and flakes (with the presence of PEG with different molecular weights or without the presence of PEG, respectively). It was shown that the spherical Y_2_O_3_:Eu^3+^ particles, obtained through a two-step spray pyrolysis process, had higher PL intensity than those with other morphologies.

In a similar work to the previous ones, also published in 2005, it was demonstrated that the densified particles of Y_2_O_3_:Eu^3+^ remarkably improved the intensity of PL emissions [100]. High luminous Y_2_O_3_:Eu^3+^ phosphor particles with spherical shape were synthesized by Spray Pyrolysis technique. A simple but effective preparation strategy for enhancing the PL intensity of these particles was implemented. The yttrium nitrate solution was modified using an organic additive, then non-hollow particles were reached, but they were very porous, and the PL intensity was not improved. To solve this disadvantage, a drying control chemical additive (DCCA) was used as a secondary additive. It was found that the surface area was greatly reduced, and the crystallite size was increased by the use of DCCA. As a consequence, densified Y_2_O_3_:Eu^3+^ particles showed great improvement in their PL emission intensity. 

The luminescent characteristics of Y_2_O_3_:Eu^3+^ (5 and 10 at.%) submicron particles, synthesized from the pure nitrate solutions at 900 °C, was also reported in 2010 [101]. The synthesis conditions (gradual increase of temperature within triple zone reactor and extended residence time) assured formation of spherical, dense, non-agglomerated particles with a crystallite size about 20 nm with a cubic Y_2_O_3_ crystalline phase. PL emission spectra were studied under excitation with 393 nm and together with the decay lifetimes for Eu^3+^ ion ^5^D_0_ and ^5^D_1_ levels revealed the effect of nanocrystalline nature on the luminescent properties of the powders. The PL emission spectra showed typical Eu^3+ 5^D_0_ → ^7^F_i_ (i = 0, 1, 2, 3, 4) electronic transitions with dominant red emission at 611 nm, while the lifetime measurements revealed the quenching effect with the rise of dopant concentration and its more consistent distribution into host lattice due to the thermal treatment. The nanostructured Y_2_O_3_:Eu^3+^ phosphors possess favorable morphological properties for applications as red phosphor in optoelectronic microdevices, for example for luminescent displays. 

Y_2_O_3_ powders doped with Yb^3+^ and co-doped either with Tm^3+^ or Ho^3+^ were synthesized and reported in 2012 [102]. These powders were processed at 900 °C using 0.1 M nitrates precursor solution and a cubic structure with space group Ia-3 was confirmed for all samples. Spherical particles with average size about 400 nm were generated with certain degree of porosity which alters their morphology during additional thermal treatment. The up-conversion emission spectra after excitation with 978 nm, as well as emission lifetimes and up-converted emission intensity dependence on excitation power were investigated. Dominant green (^5^F_4_, ^5^S_2_ → ^5^I_8_) and blue (^1^G_4_ → ^3^H_6_) emissions were found for Ho^3+^ and Tm^3+^ samples, respectively. The enhanced emission intensities and lifetime in thermally treated samples were correlated with morphological and structural changes observed. 

The enhancement of the PL emission intensity from Y_2_O_3_:Er^3+^ thin films with Li^+^ as co-dopant was published in 2013 [103]. These films were deposited using 0.03 M of yttrium acetylacetonate, dissolved in *N*,*N*-dimethylformamide. The doping of the films with Er was achieved by adding erbium (III) acetate in the solution at 1.5% in relation to the Y content. The co-doping with Li was achieved adding lithium acetylacetonate to the spraying solution; the Li contents studied were 0, 0.5, 1, 2, 3, 3.5, and 4 at.% in relation to the Y content. The films were deposited at 500 °C on (1 0 0) silicon wafers. These films were polycrystalline with a pure Y_2_O_3_ cubic phase. The typical Er^3+^ related emission spectra showed an intensity increase by a factor of ~4–5 times with the addition of 2% of Li^+^. This behavior is attributed to the distortion of the local crystalline field induced by the incorporation of Li^+^ ions. The addition of Li^+^ reduces the intensity of the diffraction peaks after 1%, and shifts the main diffraction peak toward large angles for Li^+^ doping less than 3%. The distortion of the crystalline field leads to an increment of the efficiency of intra-4f transitions by permitting the otherwise parity forbidding transitions and reducing alternative nonradiative processes. These results showed that the low-cost ultrasonic spray pyrolysis technique was a simple way to obtain rare earth doped metallic oxide films co-doped with Li^+^ ions as a strategy to improve their PL emission intensity.

The enhancement of the PL emission from Y_2_O_3_:Er^3+^ films, with the incorporation of Li^+^ ions, was reported in 2014 [104] for both visible and IR characteristic emissions of Er^3+^ ions. The presence of Li^+^ ions in the USP deposited films was inferred from Fourier transform infrared (FTIR) spectroscopy and also measured by Ion Beam Analysis (EBS), in which the high energies α particle yield from the ^7^Li(p,*α*)^4^He nuclear reaction was used to determine the content of Li^+^ inside the films. The average content of Li^+^ inside the films, as determined by EBS, increases from 0 up to 18.5 at.% for un-doped to 4 at.% Li^+^ co-doped samples. The Li-C-O absorption band in the IR region was directly proportional to the Li^+^ content inside the films and a calibration curve was generated with the EBS analysis. In a related work [105], the effect of Li^+^ co-doping on PL time decay characteristics of Y_2_O_3_:Er^3+^ was reported for films deposited at 500 °C. The Er^3+^ content, in this case, was fixed at 1.5 at.% while the Li^+^ content in the spraying solution was varied from 1 to 4 at.% in relation to Y^3+^ content. The addition of Li^+^ content up to 2 at.%, besides resulting in an increase of the luminescence emission intensity, modified the luminescence time decay behavior as well. A simple model in which charge transfer from localized centers to the Er^3+^ ions was proposed to describe the temporal evolution of the PL emission. The introduction of Li^+^ ions in Y_2_O_3_:Er^3+^ had an impact on the charge transfer (CT) process and on the total number of Er^3+^ ions contributing to the PL emission. The PL time decay characteristics of Y_2_O_3_:Er^3+^ films under 207 nm or 414 nm excitation light were analyzed with a simple model in which, in addition to the radiative recombination sites associated with Er^3+^ ions, a CT process from localized states was considered.

Luminescent and structural characteristics of Y_2_O_3_:Tb^3+^ thin films deposited from β-diketonates as precursors on c-Si substrates, at temperatures in the 400–550 °C range, were reported in 2014 [106]. The PL and CL spectra intensity depended strongly on substrate temperature, the thickness of the films and the Tb^3^^+^ doping concentration. Y_2_O_3_:Tb^3+^ thin films exhibited one main band centered at 547 nm due to the ^5^D_4_ → ^7^F_5_ electronic transition of the Tb^3+^ ion. A concentration quenching of the luminescence intensity was observed. At high temperatures the cubic crystalline phase of Y_2_O_3_ was observed as well as a reduction of organic residues. Also, at elevated temperatures, a low average surface roughness was obtained in the films with a high transmittance in the visible region.

PL and CL from Y_2_O_3_ doped with Tb^3+^ and Eu^3+^ ions films results were published in 2015 [107]. The deposition conditions were similar to those of the work described above. The optical and structural characterization of these films was carried out as a function of substrate temperature and Tb^3+^ and Eu^3+^ concentrations. Films deposited above 450 °C exhibited the typical PL bands associated with either Tb^3+^ or Eu^3+^ intra electronic energy levels transitions. The most intense PL and CL emissions were found for dopant concentration of 10 at.% for Tb^3+^ and at 8 at.% for Eu^3+^ ions in spraying solution. Higher substrate temperatures improved the crystallinity of Y_2_O_3_ films, and showed a low average surface roughness (62 Å for Y_2_O_3_:Tb^3+^_,_ and 25 Å for Y_2_O_3_:Eu^3+^ thin films). The films reported in this work were dense, and showed high refraction index (1.81), as well as a high optical transmittance in the UV-Vis range (about 90%) of the electromagnetic spectrum. These results suggest the possibility of applying those films in electroluminescent microdevices. 

Recently, in 2017, an investigation on luminescent (PL and TL) Y_2_O_3_:Sm^3+^, Li nanostructured thin films was presented [108]. XRD measurements confirmed the cubic structure of Y_2_O_3_ thin films. Li ions were successfully incorporated into the Y_2_O_3_ host lattice and it served as a sensitizer for better crystallization. The crystallites sizes are found to be ~50 nm. Surface morphology appeared as carved sculptures of particles with agglomeration. Optical absorption spectrum exhibited a prominent absorption peak at 270 nm and the corresponding energy gap was found to be ~5.53 eV. A broad PL emission was observed in the range 560–690 nm with peaks at 595, 608 and 622 nm corresponding to characteristic electronic transitions in the Sm^3+^ ions. These films were irradiated with γ-rays in a dose range 187–563 Gy; TL glow curve is deconvoluted into three peaks with temperature maxima at 400, 460 and 580 K. The activation energy and frequency factor of these TL glows were found to be in the order of ~0.58 eV and ~10^6^ s^-1^, respectively. Trap depths for the three luminescent centers were calculated and dose response was found to be linear in the range of 422–469 Gy.

### 4.5. ZnO

Zinc oxide (ZnO) is one of the most studied materials due to the various areas in which it is used. This material in the form of films and powders has also been frequently synthesized by the spray pyrolysis technique. One of the first studies on luminescent films deposited by the PSP technique of this material was on ZnO:TbCl_3_ films published in 1987 [8]. Both intrinsic and ZnO:TbCl_3_ films were deposited at atmospheric pressure, using air as the carrier gas. The substrate temperature during deposition was varied from 270 to 400 °C. The solution flow rate was changed in the range of 4–16 cm^3^/min and the carrier gas flow rate was kept constant throughout the deposition process at 10 I/min; the deposition time was 10 minutes in all cases and the TbCl_3_ concentration was 10 at.%. These films were polycrystalline with a hexagonal wurtzite structure. The PL spectra from un-doped films showed a peak centered at 510 nm [109], while ZnO:TbCl_3_ films showed a peak at 550 nm associated to electronic transitions in the Tb^3+^ ions. Later in a follow up study about these films [110] it was reported that the light emission of the ZnO:TbCl_3_ decreased with time of exposure of the sample to the excitation radiation. The phenomenon was interpreted in terms of a simple model in which a competitive process of hole trapping and photo-detrapping occurred at a radiative recombination center generated by the presence of TbC1_3_. 

The luminescence of undoped ZnO films, deposited from zinc nitrate solution, was also published in 1998 [111]. The films had a polycrystalline hexagonal wurtzite type structure with no preferred orientation. Green and orange PL (excited by 320 nm light) with emission intensity strongly dependent on the deposition and annealing temperatures was reported. The best green (broad band peaked at 510 nm) luminescent films had a porous structure while orange (band peaked at 640 nm) films consisted of close-packed grains with diameters of up to more than 1 micrometer. Green and orange PL bands resulted from oxygen-poor and oxygen-rich states, respectively, in ZnO. In the case of the green films, the vacancies did not appear to penetrate deeply into the crystallites. 

The effect of Li ions incorporation on the luminescence of ZnO films was reported in 1990 [112]. The spraying solution was 0.1 M zinc acetate in isopropyl alcohol and deionized water mixed in equal proportions. Lithium chloride was added to the spraying solution at a concentration of 10 at.%. All deposited films exhibited a hexagonal polycrystalline structure. The optical transmission depended on the deposition temperatures (Ts = 340–330 °C) which showed an absorption edge shifting to longer wavelengths with higher Ts. The PL spectra of samples deposited at low Ts showed two emissions located at 420 nm and 500 nm, associated with blue emission from the Pyrex glass substrate and the blue green emission typical of un-doped zinc oxide, respectively. Films deposited at high Ts showed an emission peak centered at 555 nm apparently associated with the localized states generated by incorporation of Li ions in the ZnO films. 

The photoluminescence from PSP deposited indium doped ZnO films was reported in 1992 [113]. This study was carried out as a function of the substrate temperature and solution flow rate. Deposition solution was 0.1 M zinc acetate in three parts of isopropyl alcohol mixed with one part of deionized water. Indium doping was achieved by adding InCl_3_ to the spraying solution in a concentration of 2 at.%. The substrate temperature was varied from 260 to 320 °C. These films were polycrystalline with a hexagonal crystalline structure; high solution flow rates resulted in larger disorder on the orientation of the polycrystallites. The PL spectra from films deposited at low substrate temperature or with high solution flow rate showed a broad peak centered at 530 nm which was associated with (In_Zn_ Vz)-luminescent centers. 

The green photoluminescence efficiency and free-carrier density in ZnO phosphor powders were investigated in 1997 [114]. An aqueous zinc nitrate solution (10 at.% Zn) was utilized in the synthesis of all powders at processing temperatures from 700 to 900 °C. Electron paramagnetic resonance, optical absorption, and photoluminescence spectroscopy were combined to characterize ZnO powders. Green PL emission was generated and a good correlation between the 510 nm green emissions with the density of paramagnetic isolated oxygen vacancies was observed. Also, both quantities increase with free-carrier concentration n_e_, as long as n_e_ < 1.4 × 10^18^ cm^−3^. At higher free-carrier concentrations, both quantities decrease. A model is proposed involving the isolated oxygen vacancy as the luminescence center. It was also shown that a free-carrier depletion layer, which forms at the surface of the powder particles, and the overall free-carrier concentration of the particles have a large impact on the green emission intensity of the ZnO powder. 

PL from ZnO and ZnO:Li films, reported in 1997 [115], showed the well-known blue-green emission typical of ZnO for the undoped films. The Li-doped films PL emission was a broad band composed of four overlapping peaks at 508, 590, 604 and 810 nm (the excitation wavelength was 365 nm); the PL excitation spectra indicated that the excitation mechanism is primarily due to electron-hole pair generation across the ZnO energy bandgap. The decay time measurements of the PL showed that the lifetime of the luminescence emission was 187 ns. The dependence of the luminescent intensity with temperature showed an activation energy of 0.057 eV for competitive non-radiative transitions. These results were indicative that the lithium was atomically incorporated giving rise to a donor level in the ZnO.

PL dependence on the deposition temperature, film thickness, and post heat treatment of ZnO films, deposited from 0.4 M solution of zinc acetate dihydrate in a mixture of deionized water and isopropyl alcohol, was reported in 2000 [116]. Chlorine free ZnO films were obtained using zinc acetate as a precursor with the (002) oriented wurtzite structure in the substrate temperature range 250–350 °C. For films with the same thickness, the intensity of green emission decreased with an increment of the O/Zn ratio as determined by XPS. The green emission intensity was gradually enhanced with increasing film thickness. Increasing deposition temperatures resulted in a reduction of the O/Zn ratio and an increment of the intensity of the green PL emission. Also, as the annealing temperature was increased, the O/Zn ratio decreased, and the green emission was consequently enhanced.

The CL from ZnO and ZnO:F (5 at.%) films deposited from ZnCl_2_ precursor solutions and fluorine doped by adding NH_4_F to spraying solution was reported in 2002 [117]. The optimal substrate temperature was 450 °C presenting a hexagonal close packed structure. The CL spectra of both ZnO and ZnO:F films exhibited near-ultra-violet band peaked at *λ* = 382 nm, but they differ on the visible emissions; the undoped ZnO films emitted an intense blue-green light at 520 nm and a red emission at 672 nm, the fluorine doped samples presented a new band emission centered at 454 nm and no blue-green emission. This emission was interpreted as coming from a lattice modification of the Zn^2+^ environment in the crystal that could be due to a total anionic substitution process of O by F species. 

Luminescent properties of ZnO and ZnO:Sn (6 at.%) films were studied through cathodoluminescence as well, in 2003 [118]. The spraying solutions (0.05 M) were prepared from Zn and Sn chlorides dissolved in deionized water. The substrate temperature was fixed at 450 °C. Luminescence films had a polycrystalline hexagonal wurtzite type structure. The CL measurements of the undoped films showed three bands centered at 382, 520 and 672 nm. Incorporation of tin ions extinguishes the blue–green band (520 nm) while appears a blue light at 463 nm and increases the value of the band-gap transition. CL imaging of ZnO films showed that the luminescence was located at defined sites giving rise to a grain-like structure inherent to the surface morphology. The presence of Sn inside the films led to great luminescent spots, attributed to large grain sizes.

The photoluminescent properties of Eu^2+^ and Eu^3+^ ions in ZnO phosphors were reported in 2004 [119]. These particles were synthesized from a zinc acetate solution and europium nitrate as the europium ions source. The crystal structure (zincite) of samples depended on the europium ions and the synthesis temperature. It was identified the coexistence of Eu^2+^ and Eu^3+^ ions in the as prepared ZnO samples. With addition of a 0.5 mol% concentration of europium ions, only the Eu^2+^ ion was detected inside the samples, while both Eu^2+^ and Eu^3+^ ions existed in samples using 1 mol% or higher concentration of europium ions. Changing the excitation wavelength, it was observed that both the blue and red PL can be obtained. The reduction of the Eu^3+^ to Eu^2+^ ions occurred in the particles prepared by the addition of a low concentration of europium ions. This reduction changed the color of PL from red to blue. Blue PL can be enhanced by increasing the synthesis temperature. At a high concentration of europium ions, the Eu^3+^ created the Eu_2_O_3_ component forming a ZnO–Eu_2_O_3_ composite.

The origin of the well-known blue-green emission of ZnO thin films was discussed on the basis of variation of the properties induced by different treatment of these films, such as ion beam irradiation (120 MeV Au ions and 80 MeV Ni ions were used for ion beam irradiation), and doping (Indium) [120]. PL studies of untreated thin films showed only one emission at 517 nm at room temperature while the irradiated films showed a decrease in this emission intensity. Indium doping also reduced the intensity of this emission; but additional emissions (centered at 407, 590 and 670 nm) were observed in these thin films. It was proposed that the blue-green emission was due to the transitions from the bottom of the ZnO conduction band to the level associated with an oxygen antisite (O_Zn_).

Photoluminescence from Er-doped ZnO films were reported in 2008 [121]. These ZnO:Er films were deposited on (1 0 0) MgO wafers at 550 °C; the concentration of Er ions in the deposition solution (from Zn and Er acetates in methanol at 0.1 M) varied from 1.0 to 3.0 at.%. The films were polycrystalline with a dominant [002] preferential orientation. The near-ultraviolet (n-UV) PL from undoped ZnO films, n-UV peaks at 3.375 and 3.360 eV were observed at 18 K, which were proposed to be originated by free excitons and donor-bound excitons, respectively. The peaks from the free exciton transitions disappeared at room temperature. However, Er-doping enhanced the room temperature n-UV emission of ZnO:Er films. ZnO:Er (2.0 at.%) films showed n-UV peaks which were ~15 times stronger than those of undoped ZnO films. 

Also, the luminescence of ZnO and ZnO:Ag nanocrystalline films deposited on Si (1 0 0) substrates from aqueous solution prepared by Zinc acetate dehydrate and Silver nitrate (6 at.%) was reported in 2008 [122]. Intrinsic samples deposited at 500 °C with spray rate of 0.15 mL/min presented the best near-band edge near-ultraviolet emission at 378 nm observed within a set of samples deposited at different deposition temperature and spray rates. The PL intensity ratio of the n-UV emission to the deep-level emission had a largest value of 470 and the full-width at half-maximum of n-UV peak had a smallest value of 10 nm (87 meV). In addition, the n-UV emission intensity of ZnO:Ag films (with the Ag:Zn atomic ratio = 3% in the precursor solution) is markedly enhanced and the ratio to the deep-level emission, increased to at least 700. However, a silver phase was detected and the n-UV luminescence became weak for ZnO:Ag films after the annealing at 700 °C in air for 1 h.

The electrical resistivity and the photoluminescence of zinc oxide films were correlated and reported in 2009 [123]. ZnO thin films were deposited, in this case, using zinc acetate dehydrate dissolved in methanol, ethanol, and deionized water within the substrate temperature range 320–420 °C. PL measurements showed that the as-grown ZnO thin films exhibited ultraviolet and green emission bands when excited by an Hg arc lamp using 313 nm as the excitation source. A red-shift in the near band edge was observed with the increase in the deposition temperature and was attributed to the compressive intrinsic stress present into the films. It is confirmed that oxygen vacancy (VO) is the most important factor that causes the broad visible emission. Furthermore, the visible emission and electrical resistivity of ZnO thin films are found to be a function of porosity. Additionally, it has been found that the intensity of the green emission at ~2.5 eV increased when ZnO films were deposited at 320 °C. The reason might be that the intrinsic stress, surface-to-volume ratio and porosity were incremented at low substrate temperatures. The resistivity presented similar behavior as the intensity of the green emission. A new luminescence mechanism based on the recombination related to oxygen vacancies in Zn-rich or stoichiometric conditions, was proposed.

Another study about ZnO:Li films was reported [124] for thin films deposited on borosilicate glass substrates; the deposition temperature was kept at 250 °C. The spraying solution was 0.2 M zinc acetate in a mixture of equal proportion of isopropyl alcohol and deionized water. Lithium doping was achieved by adding required amount of lithium acetate to the spraying solution. The spray time was 2 min with solution flow rate of 18 cm^3^·min^−1^ and gas flow rate of 15 L·min^−1^. The polycrystalline nature of the films was confirmed from XRD and TEM studies. A two-dimensional fringe moiré pattern with spacing of 1.2 nm was observed for the Li doped thin films. Lithium doping increased the roughness of the surface, thus making the film more passivated. Lithium was founded to play a key role in the excitonic as well as visible PL of ZnO films.

The effect of introducing Yb ions into ZnO films was reported in 2011 [125]. Yb-doped ZnO thin films were deposited on glass substrates at 350 °C during 77 min with a flow rate of the solution fixed at 2.6 mL/min; the molar ratio of Yb in the spray solution was varied in the range of 0–5 at.%. XRD measurements showed that the undoped and Yb-doped ZnO films exhibit the hexagonal wurtzite crystal structure with a preferential orientation along [002] direction. All films exhibited a high transmittance. The PL measurements showed a band at 980 nm that is characteristic of Yb^3+^ transition between the electronic levels ^2^F_5/2_ and ^2^F_7/2_. This was an experimental evidence for an efficient energy transfer from ZnO matrix to Yb^3+^ ions. These films showed low resistivity and high carrier mobility which makes of interest to photovoltaic devices; all ZnO:Yb thin films were n-type semiconductor. Also, ZnO:Yb^3+^ films had potential as candidates for photons down conversion process. 

An investigation of structural, optical and luminescent properties of sprayed N-doped zinc (NZO) oxide thin films was reported in 2012 [126]. The precursor solution (0.1 M of zinc acetate and *N*,*N*-dimethylformamide) was sprayed onto the preheated corning glass, and fluorine doped tin oxide substrates held at optimized substrate temperature of 450 °C. Influence of N doping on structural, optical and luminescence properties were studied. These films were nanocrystalline having hexagonal crystal structure. Raman analysis depicted an existence of N-Zn-O structure in NZO thin film. XPS spectrum of N 1s showed the 400 eV peak terminally bonded, well screened molecular nitrogen (γ-N_2_). Lowest direct band gap of 3.17 eV was observed for 10 at.% NZO thin film. The UV, blue and green deep-level emissions in PL of NZO films were due to Zn interstitials and O vacancies. The intensity of UV emission band increased with the concentration of activated nitrogen impurities. Shifting of PL peak from 393 to 388 nm seemed to be associated with free electron to neutral acceptor transition or some LO phonon replicas, followed by free electron-acceptor transitions.

The effect that Ga has on the properties of ZnO films deposited with an aqueous solution of 0.1 M zinc acetate and gallium nitrate on corning glass substrates was reported in 2012 [127]. XRD study depicted that the films were polycrystalline with hexagonal crystal structure and strong orientations along the (0 0 2) and (1 0 1) planes. Presence of E^high^_2_ mode in Raman spectra indicated that the gallium doping does not affect the hexagonal structure. The ZnO:Ga thin films were adherent, compact, densely packed with hexagonal flakes and spherical grains. Optical transmittance was high (about 80%). PL spectra showed violet, blue and green emission in these films. The specific heat and thermal conductivity study showed that the phonon conduction behavior was dominant in these films. XPS analysis confirmed that the majority Zn atoms remain in the same formal valence state of Zn^2+^ within an oxygen-deficient ZnO host lattice. The presence of zinc and oxygen vacancies was confirmed from PL results. The potential use of these films for optoelectronic microdevices was considered possible. 

Optical and structural characteristics of ZnO:Al microrod films, obtained using different solvents (methanol and propanol), were published [128]. Zinc chloride at 0.1 M concentration in methanol and propanol was used as spraying solution. The doping was achieved by the addition of Alq3 (tris(quinolin-8-olato) aluminum(III)) dissolved in chloroform with a concentration of 7 at.% Al; a 50 nm/min deposition rate on glass substrates, at 500 °C and a spray rate of about 5 mL/min, was achieved. Both undoped ZnO and ZnO:Al films were composed of microrods with hexagonal crystal structure and a (0 0 2) preferential orientation. SEM images revealed a quasi-aligned hexagonal shaped microrods with diameters varying between 0.7 and 1.3 micrometers. Optical studies showed that microrods had a low transmittance (~30%) and the band gap increased from 3.24 to 3.26 eV upon Al doping. PL measurements showed the two emission bands usually present in ZnO PL spectra: One sharp and intense peak at ~383 nm and one broadband ranging from 420 to 580 nm. 

The lithium effect on the blue and red emissions of ZnO:Er thin films was reported in 2013 [129]. These films were successfully deposited on heated (at 450 °C) glass substrates. The spraying solution was 0.05 M zinc chloride; erbium doping was achieved by adding ErCl_3_ in concentrations of 2, 3, 5, and 7 at.%. Lithium was obtained from Li_2_SO_4_ in concentrations of 3, 5 and 7 at.%. This study was an investigation of the Li effect on the enhancement of CL emission intensity on Er-mono doped ZnO films. The Li–Er co-doped ZnO films showed a higher CL intensity of blue and red emissions than the Er-mono doped ZnO films. This behavior was attributed to the modification of the local symmetry of the Er^3+^ ions, which increases the probabilities for the radiative intra 4f transition of the Er^3+^ ions to occur. These results suggested that optimized Er–Li-codoped ZnO films could be used in data storage devices.

The blue luminescence of ZnO:Zn nanocrystals prepared from zinc acetate dihydrate aqueous solutions (0.05 M), and air as carrier gas with 1, 3, and 5 L/min flow rate was also reported in 2013 [130]. The temperature of the tubular reactor was set at 500, 600, and 700 °C. The crystal sizes were about 14–22 nm with a zincite structure; the observed morphology was partially spherical with other particles of irregular shape. The highest PL intensity, peaked at 450 nm (excitation wavelength of 250 nm), was obtained from samples prepared using 5 L/min carrier gas at 700 °C. These PL emission was associated to oxygen vacancy in the ZnO:Zn nanocrystals. 

PL emission from ZnO:Ag films, formed by nanorods (NRs) as a function of the measurement temperature (10–300 K), was published in 2014 [131]. These films were deposited on soda-lime glass substrates at the deposition temperature of 400 °C and different deposition times (3, 5, and 10 min). The spraying solution (0.4 M) was prepared from zinc acetate and silver nitrate dissolved in in a mix of deionized water, acetic acid and methanol, a constant [Ag]/[Zn] ratio of 2 at.% was used for ZnO: Ag films deposition. The de position time variation permitted modifying the ZnO phase from the amorphous to crystalline, to change the size of ZnO:Ag NRs and to vary the PL emission spectra. PL spectra, versus temperature, revealed that the band related to the acceptor AgZn (LO phonon replicas of an acceptor bound exciton (2.877 eV)), and its second-order diffraction peak (1.44 eV) disappeared in the temperature range of 10–170 K with the formation of free exciton (FX). The PL intensity of defect related PL bands decreases monotonously in the range 10–300 K with the activation energy of 13 meV. The PL band (3.22 eV), related to the LO phonon replica of free exciton (FX-2LO) and its second-order diffraction peak (1.61 eV) increased in the range 10–300 K. FX related peak dominated in PL spectra at room temperature testifying the high quality of ZnO:Ag films deposited by the ultrasonic spray pyrolysis process.

A study on the role of substrates on the structural, optical, and morphological properties of ZnO films (nanotubes) was also reported in 2014 [132]. The role of substrate on the properties of ZnO films was investigated; these films were deposited onto glass, ITO coated glass and sapphire substrate and annealed at 400 °C for 3 hours. Aqueous solution (0.1 M) of zinc acetate was used to deposit these films at 350 °C. In the characterization XRD, SEM, Atomic force microscopy (AFM), and PL measurements were employed. XRD measurements showed that the ZnO films deposited on sapphire and ITO substrates exhibited a strong c-axis orientation of grains with hexagonal wurtzite structure. Extremely high UV emission intensity was observed in the film on ITO. The different luminescence behavior was discussed, which would be caused by least value of strain in the film—it is well known that the visible emission of ZnO thin films is due to the lattice defects that form deep energy levels in the bandgap. Films grown on different substrates revealed differences in the morphology. ZnO films on ITO and sapphire substrates revealed better morphology than that of the films deposited on glass. AFM images of the films prepared on ITO showed uniform distribution of grains with large surface roughness, suitable for application in dye sensitized solar cells. It was concluded that the nature of substrate had significant effect on the crystal structure, PL spectra, and morphological characteristics of the deposited ZnO films. 

A comprehensive review on the structure, optical, and luminescence properties of ZnO:RE nanophosphors, including up-conversion (UC) and down-conversion (DC) and/or down shifting PL, was published in 2017 [133]. Some of ZnO:RE nanophosphors reviewed were synthesized by spray pyrolysis technique. The interest on RE doped ZnO for UC and DC nanophosphors has been motivated by the potential application of these materials in light emitting microdevices and photovoltaic cells. The two characteristic emissions observed in ZnO at the ultraviolet and visible regions are related, respectively, to excitonic recombination and intrinsic defects. XPS data demonstrated a correlation between the visible emission and intrinsic defects in these phosphors. In the case of the DC or down shifting processes, there was simultaneous emission related to intra-f level transitions of the RE ions and defects associated transitions in the ZnO host lattice. These emissions were mainly dependent on the synthesis process, annealing temperature, and RE ion concentration; only f → f transitions of RE ions were observed in the case of the UC process. These down and up conversion RE doped ZnO phosphors were evaluated for a possible application in solid state lighting devices and photovoltaic cells. 

Also, in 2017 a study on the morphological, structural and optical properties (PL and CL) of ZnO thin films formed by nanoleafs or micron/submicron cauliflowers was reported [134]. Precursor solution was composed of zinc acetate dihydrate in deionized water (resistivity: 18 MΩ·cm); solution concentration was 0.002–0.064 mol·dm^3^ and reactor temperature was varied from 300 to 450 °C, in 50 °C steps. These films formed by nano and microstructures with hexagonal crystal phase were successfully synthesized on aluminum or silicon substrates. The morphology showed the presence of three types of particles: Nano-leafs, single microparticles, and particles formed by the aglomeration of microparticles. The largest zone was formed by nanoleafs with a width of 25 nm and a length 200 nm long regardless of the roughness of the substrate. Moreover, the energy bandgap (3.26 eV) was invariant to changes in synthesis parameters. The optical measurements showed no considerable differences between the luminescence properties of films formed by nanoleafs and cauliflower particles. Deconvolution of PL emission spectra made it possible to elucidate the existence of oxygen vacancies, interstitial oxygen, zinc vacancies and interstitial zinc, structural defects in nanoleafs, and micro-cauliflowers. Defects such as these play an important role into PL and CL emissions of ZnO because electronic transitions associated to these defects originated almost the 100% of these emissions. 

In 2018, a paper on the enhancement of visible luminescence and photocatalytic activity of ZnO:Cu thin films was published [135]. ZnO thin films doped with copper (0–4 at.%) were deposited on glass substrates maintaining a substrate temperature of 400 ± 10 °C. 0.4 M solution of zinc acetate and cupric acetate dissolved in a mixture of methanol, deionized water and acetic acid was used as the precursor for the deposition of these thin films. Hexagonal crystallinity (wurtzite) of the films improved at lower doping concentrations due to the easy fitting of Cu dopants in the Zn host lattice sites and preferred highly textured growth along the (0 0 2) plane. Higher doping concentration deteriorated the crystallinity and the optical transmission. EDX measurements confirmed the incorporation of Cu in the doped films. Optical energy gap red-shifted with the addition of Cu contents due to the exchange interactions and difference in iconicity of Zn and Cu. Cu doped films exhibited strong PL visible emission due to the modulation of the band structure and subsequently new levels acting as emission centers were formed in the forbidden bandgap of ZnO films. The addition of Cu ions increases the concentration of zinc interstitials, as well as zinc and oxygen vacancies which cause more intense emission in the visible region. ZnO:Cu thin films exhibited very good photocatalytic activity due to the efficient trapping of photo-generated electrons thereby suppressing the electron-hole recombination and higher doping level slightly decreased the degradation efficiency because excess dopants may act as recombination centers.

The effect of fluorine and boron co-doped ZnO thin films on the structural and luminescence properties was published in 2018 [136]. Fluorine and boron co-doped zinc oxide (ZnO:B:F) thin films were deposited on the corning glass substrates at 400 ± 5 °C: The spraying solution was prepared by mixing zinc acetate, boric acid, and ammonium fluoride, dissolved in methanol and deionized water with a ratio of 3:1. After characterization it was found that ZnO:B:F films had high average optical transmittance; XRD patterns indicated that the obtained ZnO:B:F films had a hexagonal wurtzite type structure with (0 0 2) preferential orientation. The crystallite sizes were in the 18–40 nm range. Green emission and UV emission band are observed in PL spectra of ZnO and ZnO:B:F. Undoped ZnO films exhibited only one peak around 390 nm associated with near band ultra violet emission. It is well known that the UV emission peak usually originates from the near band-edge emission from the recombination of free exciton. Also, it was considered that the intensity ratio of UV to visible emission is commonly considered as a sign of perfect crystal quality and low defect concentration. A green emission peak was observed for ZnO:B:F films; the intensity of this peak centered at 520 nm increased while the B–F concentrations increased. The observed green emission is also due to the impurity levels related the oxygen vacancy (Vo) in ZnO:B:F films. The electrical resistivity, carrier concentration and Hall mobility also were measured. The highest Hall mobility of 13.22 cm^2^ v^−1^·s, and the lowest electrical resistivity of 3.13 × 10^−4^ Ω·cm, were obtained at the optimal boron-fluorine co-doping concentration of 5 at.%. All of the results were appreciated in point of view of optoelectronic industry and photovoltaic solar cell applications and it was concluded that B-F co-doping has a positively effect on electrical properties.

### 4.6. ZnS

A research on the luminescence of ZnS, ZnS:TbC1_3_ and ZnS:SmC1_3_ films, deposited by the Pneumatic SP technique, was first reported in 1988 [137]. The ZnS films were deposited using a spraying solution obtained by mixing in equal proportions solutions of 0.1 M of Zn acetate and 0.1 M of dimetylthiourea (C_3_H_8_N_2_S), both dissolved in three parts of isopropyl alcohol and one part of deionized water. The doped films were prepared by adding TbCI_3_; or SmCI_3_, to the spraying solution at a 10 at.% concentration; the substrate temperature, during the deposition, was either 300, 330, 360, or 375 °C. The solution flow rate was 14 cm^3^/minute and the nozzle substrate distance was 30 cm in all cases. The doped films exhibited strong PL emission with a blue dominant peak at about 460 nm. This peak is characteristic of chlorine-doped ZnS phosphors. The films had poor crystallinity with a cubic crystalline structure. The optical transmission (T about 80%) characteristics of these films showed an absorption edge shifted to shorter wavelengths compared with those of the undoped films. Photoluminescent characteristics of In-, Al-, and Cu-doped ZnS films were reported in 1989 [138]. These films were deposited from a spraying solution formed by 0.1 M (CH_3_COO)_2_ + 0.1 M C_3_H_8_N_2_S dissolved in a mixture of three parts of isopropyl alcohol plus one part of deionized water. Doping was reached adding InC1_3_, A1C1_3_, or CuCl to the starting solution. The substrate temperatures were either 270, 300 or 330 °C. The substrates were pyrex glass slides, pyrex glass coated with In_2_O_3_ and silicon oxide. All films showed polycrystalline features which could be associated to a wurtzite structure of ZnS. Also, the presence of chlorine was detected into the films in quantities that depended on the deposition parameters. The PL spectra measured at room temperature displayed different emission peaks for each one of the impurities. The PL spectra from the Al-doped ZnS films showed a peak centered at 470 nm. The In-doped ZnS films showed a peak about 545 nm and the PL spectrum from the Cu-doped films exhibited a peak at 570 nm. The shape and intensity of the PL spectra do not depend strongly on the type of substrate.

The luminescent properties of ZnS:Mn films deposited by the pyrolysis spray technique on glass substrates at atmospheric pressure using air as a carrier gas were reported, for the first time, in 1992 [139]. The spraying solution in this case consisted of 0.1 M of Zn acetate and 0.1 M of dimethylthiourea in a mixture of three parts of isopropyl alcohol and one part of deionized water. The Mn doping was achieved by mixing MnCl_2_ (0–20 at.%) in the spraying solutions; the deposition temperature was varied between 340 and 500 °C in steps of 20 °C. All films resulted polycrystalline with a wurtzite (hexagonal) structure. The PL spectra show, besides the characteristic light emission associated with Mn (yellow at 590 nm) in a ZnS host lattice, a peak associated with the self-activated emission (blue at 490 nm) observable at low substrate temperatures and/or long deposition times. The presence of chlorine impurities in the films was suggested to be associated with this emission. The Mn related luminescence showed a quenching effect with the Mn concentration (at concentrations higher than 3 at.% Mn in the spraying solution). The light emission at this center had an activation energy of 0.71 ± 0.05 eV with the deposition temperature. This energy was proposed to be related with the energy required for the Mn atoms to find a proper site during the growth process to form a Mn^2+^ center. These films were incorporated in a Metal-Insulator-active layer- Insulator-Metal (M-I-S-I-M) structure and their electroluminescent features were reported in 1995 [14]. These alternating current electroluminescent thin film structures were prepared using, for the first time, high-quality SiO_2_ insulating thin films and spray pyrolyzed ZnS:Mn^2+^ as the active layer. The structures prepared with 60 nm thick insulating films showed threshold voltages of 30 V (rms) and saturation voltages of about 56 V (rms). The electroluminescent emission spectra presented a peak centered at 590 nm (yellow emission) associated with the Mn^2+^ center. The brightness-voltage characteristics were typical for a structure of the M-I-S-I-M type. The external efficiency calculated from the charge-voltage characteristics had a value of 1.8 Lumen/Watt.

Spray pyrolysis synthesis of ZnS nanoparticles (sub-10 nm) from a single-source precursor was published in 2009 [140]. Here, it was reported the synthesis of cubic ZnS nanoparticles from a low-cost single-source precursor in a continuous spray pyrolysis reactor. In this study, a single-source precursor: Diethyldithiocarbamate, [(C_2_H_5_)_2_NCS_2_]_2_Zn, dissolved in toluene was used to synthesize ZnS nanoparticles. The furnace setpoint temperature was typically 600–800 °C. In this method, the evaporation and decomposition of precursor and nucleation of particles occur sequentially. XRD indicated a Cubic ZnS (zinc blende) for the synthesized particles. High Resolution Transmission Electron Microscopy (HRTEM) images showed ZnS particles with diameters ranging from 2 to 7 nm were. As-synthesized ZnS nanoparticles (excited at 350 nm) exhibited blue photoluminescence near to 440 nm had quantum yields up to 15% after HF treatment. This demonstrated a potentially general approach for continuous low-cost synthesis of semiconductor quantum dots, and applications in solar cells, lasers and displays. Also, ZnS nanoparticles can be applied as phosphors, probes for bio-imaging, emitters in light emitting diodes and photocatalysts.

## 5. Conclusions

This review describes some of the very extensive research work about the spray pyrolysis technique, which without doubt, is an extraordinarily flexible and practical materials synthesis method. It is a low-cost, non-vacuum required, way to synthesize materials in the form of powders and films deposited over a wide variety of substrates, and can be easily adapted for large area deposition and industrial production processes. The present work has been limited to review several luminescent materials and those with high-*K* dielectric properties, most of them metal oxides, synthesized by this process. Concerning the dielectric materials, it has been focused on the work carried out in high-*K* dielectric films of aluminum oxide, yttrium oxide, and zirconium oxide, developed for application on MOSFET technology devices. Through the works reviewed, the spray pyrolysis technique has been proved to be a technique capable of producing films as thin as 30 nm on silicon wafers with an outstanding dielectric and optical qualities, which could also be considered for design and development of sensors and other multilayered microdevices—such as planar waveguides and resonant optical structures. In the case of luminescent materials (PL, CL, TL, Up-conversion), the information reviewed shows that metal oxides (ZrO_2_, HfO_2_, Al_2_O_3_, Y_2_O_3_, ZnO) and ZnS doped with rare earth and transition metal ions with specific luminescent characteristics could be tailored according to light emitting devices, and to many other applications requirements.

## Figures and Tables

**Table 1:** Static dielectric constant of a few gate dielectrics.

Oxide	K
SiO_2_	3.9
Si_3_N_4_	7
Al_2_O_3_	9
Ta_2_O_5_	22
TiO_2_	80
ZrO_2_	25
HfO_2_	25
HfSiO_4_	11
La_2_O_3_	30
Y_2_O_3_	15
a-LaAlO_3_	30

**Table 2:** Physical properties of a few solvents used during the deposition of some high-*K* dielectrics.

Solvent	Boiling Point (°C)	Viscosity at Room Temperature (mPas)	Density (g/cm^3^)	Chemical Formula
Dimethylformamide	153.0	0.80	0.95	C_3_H_7_NO
Methanol (Methyl Alcohol)	65.0	0.52	0.79	CH_3_OH
Ethanol (Ethyl Alcohol)	78.5	1.19	0.78	CH_3_CH_2_OH
Propanol (n-Propyl Alcohol)	97.4	2.25	0.80	CH_3_(CH_2_)_2_OH
Butanol (n-Butyl Alcohol)	117.0	2.95	0.80	CH_3_(CH_2_)_2_CH_2_OH
